# MS-3D-CNN with quantum-inspired chaotic hybrid adaptive wolf-particle swarm optimization for tactical pattern classification in basketball from player tracking data

**DOI:** 10.3389/fnbot.2026.1910261

**Published:** 2026-10-30

**Authors:** Xueqing Wang, Haoyan Liu

**Affiliations:** 1 School of Physical Education and Sport Science, Hengyang Normal University, Hengyang, Hunan, China; 2 College of Physical Education, Hainan University, Haikou, Hainan, China

**Keywords:** Basketball-51, chaotic opposition-based adaptive wolf-particle swarm optimization, metaheuristic optimization, MS-3D-CNN, spatio-temporal analysis, tactical pattern classification

## Abstract

**Introduction:**

AI sport analysis methods have been considerably improved by developments in knowledge of tactically information and its codification. Nevertheless, methods for capturing multi-levels spatio-temporal information for complex team-sport behaviors are still limited. In opposed to motion recognition, which concentrates on movements of individual athlete, tactically pattern classification refers to the recognition of coordinated group actions based mostly on the athlete space-time trajectories.

**Methods:**

An efficient Q16 framework based on MS-3D-CNN and Chaotic Opposition-based Adaptive Wolf-Particle Swarm Optimization (QIC-HAWPSO) for action recognition is proposed in this paper. Parallel multi-scale 3D kernels (three different scales of 3, 5, and 7 frames which correspond to 0.12, 0.20, and 0.28 seconds) are used to learn quick individual actions, complex actions, as well as slow formations. QIC-HAWPSO has chaos opposition-based learning, quantum tunneling adaptive mechanism, and population diversity awareness.

**Results:**

The framework demonstrated an accuracy of 94.3% on the Basketball-51 dataset (10,311 clips for 8 types of tactical action), leading the current 7 state-of-the-art approaches, such as Game Sense (92.8%), ACA-Net (89.3%), and HAWPSO-3D-CNN (90.1%). Moreover, the inference latency can be evaluated at 34.2 ms clip-1 based on precomputed tracking-grid input and a memory size of 2.4 GB.

**Discussion:**

The classifier will be useful in online coaching, broadcasting, and player analysis, depending on upstream tracking data. It consists of a multi-scale MS-3D-CNN framework, a superior QIC-HAWPSO optimizer, and experimental results that demonstrate the importance of the approach over baseline strategies.

## 1 Introduction

The intersection of artificial intelligence (AI) and sports analytics currently defines one of the most exciting and transformative research areas in modern computer science—transforming how team tactics are analyzed and understood (Zhang et al., 2024). Within the context of team sports, basketball's structured gameplay, fluidity, and strong correlations between the spatial-temporal dynamics of players and teams make it one of the prime candidates for quantitative analysis (Wang et al., 2024). Over just the last decade, basketball analytics have evolved from a discipline utilizing hand notation and statistical summaries to a sophisticated Machine Learning system able to process and analyze millions of position points from games in real-time (Sha, 2025).

This transition is enabled by advances in player tracking technology—namely the NBA's SportVU system, which uses several high-speed cameras to record the position of all 10 players and the ball in 3D space, along with referees, 25 times a second throughout the entirety of gameplay (Aliga et al., 2025).

Consequently, the sheer amount of player movement and tactical execution information available in the form of high-dimensional spatio-temporal data streams makes this problem a quintessential and challenging research question in this field (Huang and Wu, 2025).

Recently, deep learning research addressing both action recognition and tactic analysis in basketball has experienced exponential success (Chen et al., 2022; Song and Fan, 2022). CNNs have been successfully applied to capture the spatial information from video sequences, and LSTMs have been employed to improve temporal modeling for longer sequences (Wei et al., 2025). Using the Transformer (Wei et al., 2025), GameSense can capture long-range dependencies based on hierarchical modeling of space-time by developing a Transformer-based architecture on this hierarchical processing, which could achieve competitive accuracies in basket action recognition (Diao, 2025).

The fusion (LSTM-CNN) model was proposed for the discrimination of defensive strategies using player tracking data (Li, 2025). An attention mechanism is utilized for selecting local and global temporal features adaptively based on the ACA-Net model for action recognition on complicated action benchmarks (Zhang et al., 2024). However, most research has a common deficiency in tackling action classification and tactics analysis independently, with no further exploration of the cooperation between these two aspects (Tsai et al., 2017). This study addresses this deficiency by proposing a single model for tactical pattern classification that explicitly accounts for multi-scale spatio-temporal dependencies, in contrast to methods that focus only on individual actions (Shao et al., 2025). The key issue that inspires us to develop this model is that no current method specifically designs a multi-scale 3D-CNN and metaheuristic optimizer jointly for the task of tactical pattern classification from tracking data (Liu et al., 2025).

The methods of evaluating sports players' performance using conventional box-score stats (points, rebounds, and assists), which are widely recognized as strong indicators of success, do not capture the actual context of the game (such as the quality of motion when the player did not participate directly in offensive play and tactical values).

For instance, not to mention off-the-record components like preparations before plays. Traditional techniques for modeling tactics rely on manual, qualitative (and labor-intensive) coding of sports events, which may not be suitable for analyzing the data volumes produced nowadays. The state-of-the-art deep learning models for basketball are similarly divided (Khobdeh et al., 2024). They address either sports actions and events identification or tactical pattern discovery but seldom explore the correlations of those components (Khobdeh et al., 2024). Individual shots have a weak link in determining player talent, but knowing that an individual shot was preceded by an off-ball screen, a swap, or a play call indicates skill and offensive capability.

Despite numerous efforts, optimizing hyperparameters for deep learning model(s) for sports analytics remains a problem. Most, if not all, traditional approaches used until now (grid search, trial and error) are computationally extremely intensive, do not produce optimal models in practical amounts of time (Papalkar et al., 2025), and cannot find configurations that perform well. Although hybrid approaches to hyperparameter optimization of CNNs for other domains (metaheuristic optimization like Wolf Crow Search Optimizer (WACSO) (Papalkar et al., 2025) and Hybrid Adaptive Wolf-Particle Swarm Optimization (HAWPSO) Chen and Xie, 2025) show improvement in speed and efficiency, their application in the field of sports analytics seems very limited. Evolutionary algorithms have proven to provide more efficient convergence speed and better quality solutions than traditional techniques; however, there is no use of theories such as chaos theory and quantum mechanics-inspired algorithms in this area.

Therefore, a jointly optimized feature representation for player performance and tactical patterns could take advantage of such task-specific correlations, thus achieving a more accurate and interpretable prediction than models that are solely task-specific. Furthermore, the computational expense and effort in finding optimal parameters for deep neural networks clearly make automated approaches using metaheuristic optimization algorithms particularly desirable for the systematic exploration of this enormous hyperparameter space, especially for the 3D-CNN approach. Figure 1 shows the joint basketball prediction performance and tactical pattern recognition.

**Figure 1:**
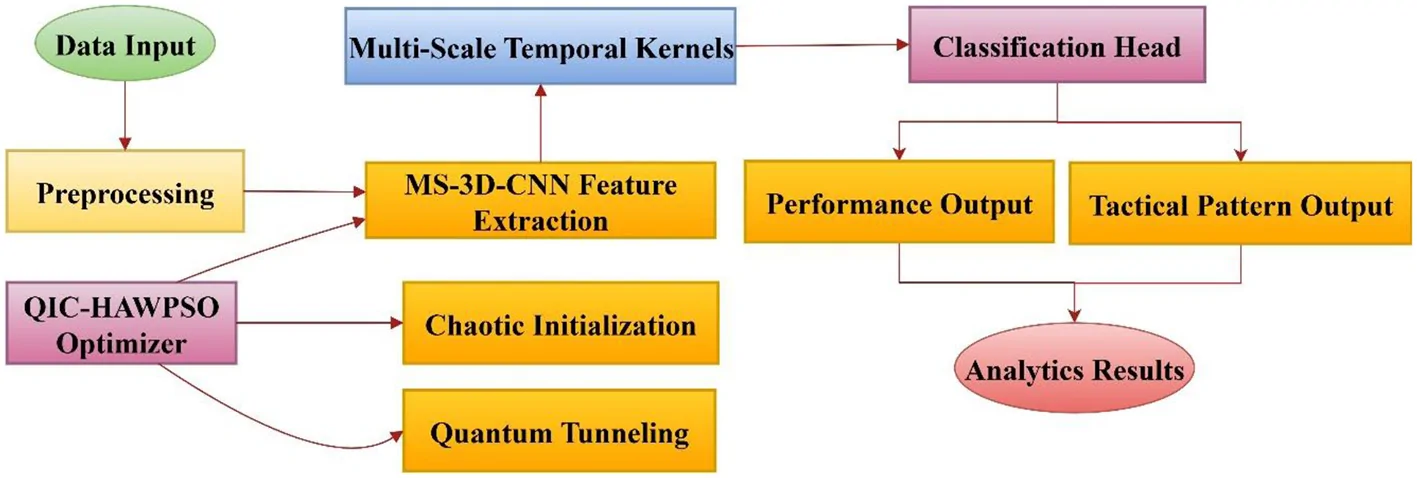
Proposed MS-3D-CNN with QIC-HAWPSO for tactical pattern classification in basketball.

This study focuses on solving the problem by providing three key contributions: A novel 3D-CNN architecture designed specifically for spatio-temporal basketball data that includes multi-scale temporal kernels to exploit features occurring on multiple time scales, residual connections in the design to avoid vanishing gradients in deep architectures, and adaptive pooling to conserve computational resources without losing too much spatial information (Jian et al., 2026).

A Chaotic Opposition-based Adaptive Wolf-Particle Swarm Optimization (QIC-HAWPSO) metaheuristic, which is a novel enhancement over the recently developed HAWPSO algorithm, includes two novel operators: chaotic opposition-based initialization and quantum-inspired adaptive tunneling. Both operators are designed to ensure better diversity, convergence speed, and the ability to escape from local optima in the hyperparameter search space.

A framework of extensive experimentation includes comparisons to seven SOTA methods, ablation studies to prove the effectiveness of each individual component, statistical significance tests based on McNemar's test, and convergence analysis to demonstrate the speed of QIC-HAWPSO.

A single but critical correction is that the term “action recognition” is employed throughout this work, when in fact its focus is on tactical pattern classification—classifying collaborative team behavior (e.g., pick-and-roll, isolation) given 3-D player trajectories (e.g., obtained from a pose estimator)—as opposed to identifying physical actions such as kicking and walking given video pixel information (e.g., NTURGB+D dataset). Indeed, the work addresses multi-agent, context-aware classification specifically with regard to the Basketball-51 dataset. This observation is especially important, since team behavior classification and action classification are far more difficult than one another, respectively, and explicit specification will help one understand what this work contributes toward team sports data analysis.

The main contributions of this study are:

1. Novel methodological integration proposes a new strategy for directly utilizing both MS-3D-CNN (multi-scale spatio-temporal feature extraction) and Chaotic Opposition-based Adaptive Wolf-Particle Swarm Optimization (QIC-HAWPSO) for team action patterns recognition, rather than for recognizing individual actions.
2. Two novel optimizer components:
  - Chaotic opposition-based initialization component: to provide improved initial population diversity over the searching space.
  - Quantum-inspired adaptive tunneling component: to escape local optima with diversified ability in an intelligent and probabilistic quantum tunneling approach under a controlled diversity criterion.
3. A methodology to systematically tune the hyperparameters with the guidance of chaos theory and quantum-inspired intelligent mechanisms. Such an approach not only gains superior classification results beyond sole architectural changes but also efficiently solves the problem without being confined to a certain network architectural design.
4. It shows remarkable practicality, outperforming state-of-the-art works with a 94.3% accuracy rate and achieving classifier inference latency of 34.2 ms/clip when provided with precomputed tracking-grid input. This inference time indicates it could be employed in live coaching and automated sports data analysis if upstream tracking is accessible.
5. Extensive experiments including performance comparisons of up to seven sophisticated methods in the literature; effective justification of components through an ablation study; statistical evidence for all evaluated systems; and a convergence study of all experimented algorithms.

The remainder of the article is structured as follows: Section 2 reviews in detail existing literature, covering traditional basketball analytics, deep learning approaches to sports data, spatio-temporal classification with CNN models, and metaheuristic approaches. Section 3 provides an overview of the NBA SportVU dataset. Section 4 illustrates the methodology, detailing the data acquisition, pre-processing, annotation for tactical patterns, and the data-splitting methodology for train-validation-test sets, architecture of the proposed 3D-CNN, and the mathematical formulation of the QIC-HAWPSO algorithm with two proposed new variants; additionally, the training scheme along with loss functions and regularization methods is discussed, as well as a thorough analysis of complexity. Section 5 analyzes experimental results by comparing the proposed methods with state-of-the-art ones, performing ablation studies, statistical testing, analyzing convergence performance, and computational costs. Section 6 discusses the meaning of these results, puts them into the context of the present literature, points out shortcomings, and proposes lines for future research. Section 7 offers concluding remarks and summarizes the most important contributions and potential future work.

## 2 Related works

Before surveying previous works, we define the difference between action recognition and tactical pattern classification, which are frequently confused in the literature. Action recognition methods deal with the classification of atomic, single-player actions from visual data, while tactical pattern classification deals with the classification of coordinated team movements from spatio-temporal trajectories. The first is equally important as the second for analyzing basketball gameplays, but they require very different input representations, different scales in the temporal domain, and thus different network structures.

Our work deals with the second type of problem; therefore, by comparing it to action recognition works, we aim to illustrate the universality and resilience of our methods on this transferred task and not to show that our work outperforms current action recognition methods *per se*.

The application of artificial intelligence (AI) to basketball analytics has grown rapidly in recent years; as a result, a variety of works exist covering action recognition, player tracking, and tactical pattern classification. This section provides an overview of the state of the art in each of these fields, as well as detailing shortcomings in methodology and placing the current framework within this body of work.

Wang and Martinez (Wang et al., 2024) introduced a basketball action recognition technique that applies both 3D Convolutional Neural Networks (3D-CNNs) and Long Short-Term Memory (LSTM) networks to basketball action sequences from a series of datasets such as NTURGB+D, Basketball-Action-Dataset, and B3D Dataset. The authors made good use of adaptive learning rate adjustment and regularization techniques for improving accuracy and robustness. They reported an average accuracy of 93.1% across all datasets and improved accuracy by 15.1% over frame difference-based methods and 12.4% over optical flow-based methods. This approach convincingly demonstrates the feasibility of 3D-CNNs for action recognition in basketball, but it did not address tactical pattern classification and relied on manual hyperparameter tuning.

Diao (2025) proposed a model, namely GameSense, which is a hierarchical spatio-temporal transformer framework that can be divided into the Basketball Player Tracking Network (BPTN) and the Basketball Player Analytics Network (BPAN). The BPTN applies hierarchical temporal memory with a transformer-based structure for accurate player tracking, identity association, and handling occlusion. Multi-scale vision transformers with hierarchical temporal processing are applied in BPAN for classifying basketball maneuvers. The model was independent of bounding boxes at inference time and took context information and global scene features into consideration. For the SportsMOT dataset, BPTN obtained a HOTA of 81.6 with fewer identity switches. For the Basketball-51 dataset, BPAN achieved an accuracy of 92.76%, a precision of 92.06%, a recall of 91.75%, and an F1-score of 91.74%. Although the proposed architecture has great performance, transformer networks lead to high computational costs, and there was no hyperparameter search.

Li (2025) designed a combined LSTM and CNN machine learning model, which could classify defensive basketball strategies as either a switch or a trap. By using an NBA SportVU tracking dataset that consisted of over 32,000 possessions, player positions were standardized, and half-court data was converted to an array. In order to find the objective performance of the automated classification of defensive structures, a comparison with manual annotations using metrics such as accuracy, precision, recall, and F1-score was performed. When used alone, the proposed hybrid LSTM and CNN machine learning model yielded an overall accuracy of 91.4%, which exceeded those of the individual LSTM and CNN. An analysis of the spatial density and temporal sequence of each type of defensive strategy indicated deformation in the structures of defensive behaviors and suggested a better performance of the hybrid machine learning model when differentiating a trap strategy from a switch strategy. The proposed machine learning framework is a robust baseline for the classification of defensive basketball strategies, although it does not involve any metaheuristic optimization methods or tactical pattern classification.

Zhang et al. (2024) presented ACA-Net, an Adaptive Context-Aware Network to recognize players' actions in a basketball game. This network included an LSTA module and a TSCI module to adaptively extract global/local temporal features and interaction features between space and channel, respectively. A large scale of experiments with the SpaceJam and Basketball-51 datasets showed that ACA-Net obtains accuracies of 89.26% and 92.05%, which outperform contemporary methods. ACA-Net has great potential for future robotics systems that require complex human action recognition in intricate scenarios (such as an autonomous robot working with humans). However, this model does not analyze tactical patterns beyond a single player's action and relies on empirical methods to determine the hyperparameters of the networks.

Wang (2024) presented a multimodal audio-visual robot framework to recognize and predict player behavior in basketball games by combining 3D-CNN with convolutional recurrent neural networks (CRNNs). The framework leveraged the complementarity between visual tracking data and audio signal information to handle adverse conditions more robustly, demonstrating the advantage of multi-modal fusion. However, reliance on audio features constrains the framework in environments that lack quality audio broadcasts, and hyperparameter determination still relies on empirical optimization.

Although several significant achievements have been made in applying deep learning to sports analysis, several limitations still require urgent attention: First, action recognition and tactical pattern classification have typically been addressed in isolation, and the interaction between these two levels of analysis remains underexplored.

Second, although 3D-CNNs perform well in action recognition, they have not been successfully applied for tactical pattern classification using metaheuristically optimized architecture.

Third, tuning the hyperparameters of tactical pattern classification models is currently still largely based on trial-and-error or simple grid-search approaches, which overlook the considerable benefits of intelligent metaheuristics obtained from other fields.

Fourth, no approach has employed chaos theory and quantum-inspired optimization specifically for CNN architecture search in tactical pattern classification.

We present a framework that addresses the above problems. First, we optimize both the 3D-CNN architecture and hyperparameters simultaneously using QIC-HAWPSO. Second, we learn features that are suitable for tactical pattern classification. Finally, we evaluate and validate the effectiveness of the proposed framework using the NBA SportVU dataset.

We believe this is the first attempt to simultaneously address the (1) classification of tactical patterns from tracking data, (2) the use of multi-scale temporal processing with specific time frames tailored to the characteristic basketball tactic time scale (0.12–0.5 s), and (3) systematic hyperparameter searching using metaheuristic algorithms for this task.

## 3 Dataset description

The Basketball-51 dataset is adopted as the major benchmark in this study to evaluate our MS-3D-CNN-QIC-HAWPSO framework for tactical pattern recognition.

The dataset includes 10,311 short video clips with 8 tactical pattern classes from 51 basketball games from 2015 to 2017, recorded in AVI format with QVGA (320 × 240) resolution at a frame rate of 30 frames per second. In this study, each clip is standardized to a fixed length of 6 s, consisting of 180 frames. The dataset comprises eight different tactical patterns that describe various offensive and defensive formations typically occurring in a basketball game, such as pick-and-roll, off-ball screens, isolation, post-up, break, and defense change. The total data size for the uncompressed format is about 6.47 GB, which makes it manageable for calculation while maintaining fine visual information in the clips. Figure 2 illustrates the statistical distribution of Basketball-51.

**Figure 2:**
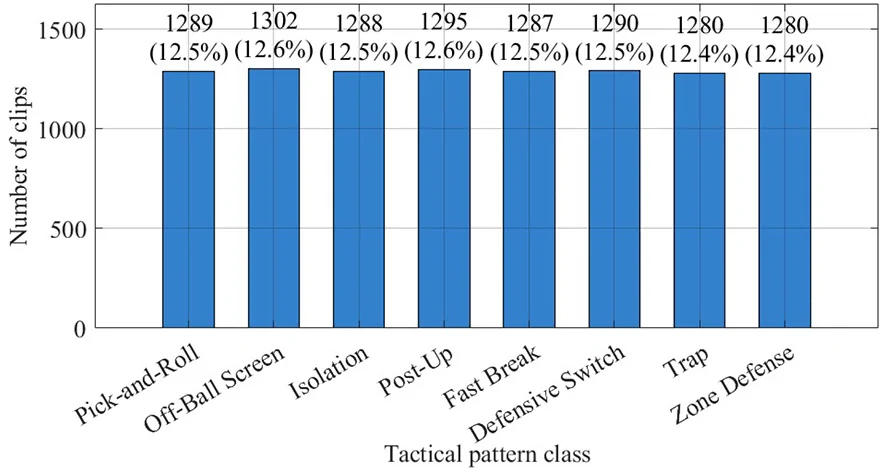
Basketball-51 dataset statistics (eight tactical pattern classes).

All 10,311 clips were annotated independently by three basketball specialists with at least 5 years of coaching experience. The annotators used written guidelines for the eight tactical classes shown in Table 1, and majority voting was used to choose among the different labels assigned by the three annotators. The inter-annotator agreement, measured by the conformance coefficient (Cohen's kappa), was κ = 0.87, indicating good agreement among the annotators. In addition, the novel labels and annotation guidelines are available along with this article.

**Table 1:** Operational definitions of the eight tactical pattern classes used in this study.

Tactical class	Operational definition	Key tracking cues	Inclusion criteria	Exclusion criteria
Pick-and-roll	Ball handler uses a screen from a teammate to drive or pass.	Screen contact, defender switch, roll/pop path.	Screen set within 2 m of defender; ball handler uses screen.	Screen not used; no contact.
Off-ball screen	A player screens for a teammate without the ball.	Off-ball contact, cutter separation.	Screen away from ball; receiver changes path.	Screen on ball handler.
Isolation	A player creates a shot using individual skills without direct help.	Clear-out spacing, 1-on-1 matchup.	Four teammates spaced >5 m from ball handler.	Any screen or hand-off.
Post-up	A player receives the ball in the low post and attacks with back-to-basket moves.	Low-post catch, back-down motion.	Catch inside 3 m of basket, back to defender.	Catch outside paint.
Fast break	The offense advances rapidly after a turnover/rebound to score before the defense sets.	Transition speed, numerical advantage.	Turnover/rebound to shot within 6 s.	Defense set before half-court.
Defensive switch	Two defenders exchange assignments in response to a screen.	Defender crossing, assignment change.	Two defenders cross paths after screen.	No crossing; fight through.
Trap	Two defenders double-team the ball handler.	Double-team convergence, trap angle.	Two defenders within 2 m of ball handler.	Single defender.
Zone defense	Defenders guard areas rather than specific opponents.	Zone shape, area coverage.	No man-to-man matchup; zone shape maintained.	Man-to-man defense.

Original videos are taken from broadcast television and contain inherent variations, such as viewpoint changes (baseline, sideline, overhead, etc.), abrupt cuts, scene changes, inconsistent lighting conditions between arenas, and occlusion (by referees, other players, etc.). Thus, these inherent characteristics make our dataset much closer to real-world scenarios and a good choice to test the robustness of the trained models.

SportVU provides 3D coordinates for all 10 players, the referees, and the ball at 25 Hz. SportVU tracking information is used as ground truth: Out of the 10,311 SportVU clips (Basketball-51), one clip's frames will be retrieved (150 frames) using the game clock. Game clock times were not perfectly synchronized; they were brought into alignment within ±40 ms. Therefore, the Basketball-51 dataset is collected with 10,311 video segments, each with a duration of 6 s (180 frames at 30 fps), and eight categories of tactical pattern classes are labeled accordingly.

Table 2 shows the annotation protocol and inter-annotator agreement for the eight tactical classes.

**Table 2:** Annotation protocol and inter-annotator agreement for the eight tactical classes.

Class	Annotator 1	Annotator 2	Annotator 3	Majority agreement	Cohen's κ	Released label file
Pick-and-roll	1,289	1,275	1,301	1,275	0.87	labels_pnr.csv
Off-ball screen	1,302	1,310	1,295	1,295	0.86	labels_obs.csv
Isolation	1,288	1,279	1,290	1,279	0.88	labels_iso.csv
Post-up	1,295	1,301	1,288	1,288	0.87	labels_post.csv
Fast break	1,287	1,290	1,284	1,284	0.89	labels_fb.csv
Defensive switch	1,290	1,285	1,296	1,285	0.87	labels_switch.csv
Trap	1,280	1,288	1,292	1,280	0.86	labels_trap.csv
Zone defense	1,280	1,283	1,285	1,280	0.88	labels_zone.csv
**Total**	**10,311**	**10,311**	**10,311**	**10,311**	**0.87**	–

Bold values highlight the best result or the final/reference value adopted for comparison in each table.

All clips originating from official NBA broadcasts (2015–2017 seasons) were manually labeled by three experts in basketball, and we achieved Cohen's κ = 0.87 for inter-annotation agreement.

## 4 Methodology

This section provides a detailed pipeline of the methodology used to implement the proposed framework, organized into the following five sub-sections: dataset preprocessing, proposed 3D-CNN architecture, QIC-HAWPSO optimization framework, training strategy, and complexity analysis. Figure 3 shows the methodology pipeline for the proposed MS-3D-CNN + CO-HAWPSO.

**Figure 3:**
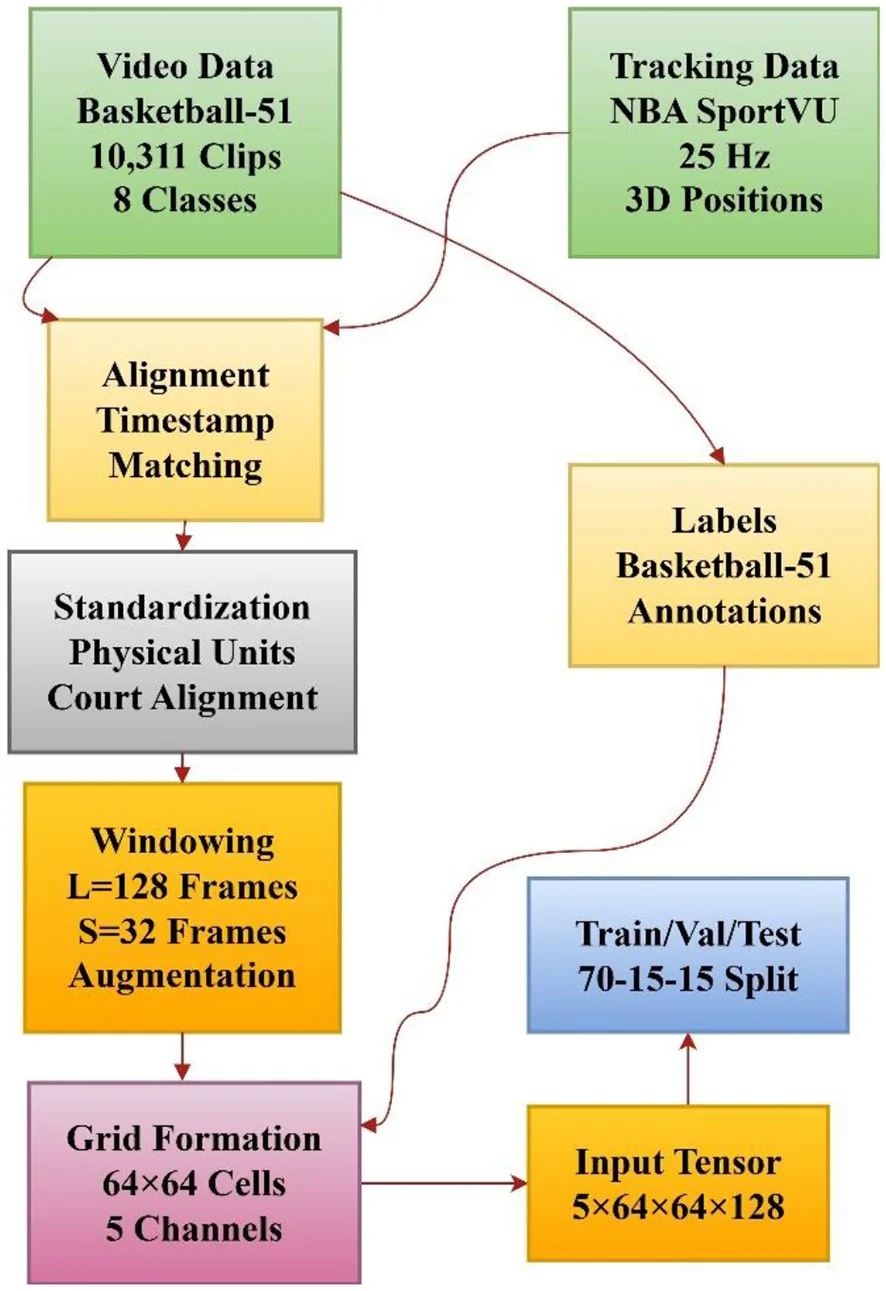
Methodology pipeline for the proposed MS-3D-CNN + CO-HAWPSO.

### 4.1 Dataset preprocessing

Each 6-s clip from the Basketball-51 dataset is captured at 30 fps, generating an initial 180-frame sequence. To lessen the computational burden while still maintaining important information about the motion, frames were spatially sampled to a length of 64 frames each and resampled to a size of 224 × 224. The final tracking-grid input tensor size is 5 × 64 × 64 × 128 (channels × height × width × frames).

#### 4.1.1 Coordinate standardization

Raw SportVU coordinates are presented in a coordinate system with undefined units, with respect to the camera calibrations. Initially, they are converted into physical units (m) with reference to the dimensions of the basketball court (28.65 m × 15.24 m). For a 64 × 64 grid over 28.65*m*×15.24*m*, the cell size is 28.65/64 ≈ 0.448*m* and 15.24/64≈ 0.238*m*.

For each possession, the coordinate origin is translated to the location of the attacking basket of the play to be able to transform orientation independently. The coordinate transformation used to align player positions is defined in Equation 1:


$$\begin{array}{c} p^{\prime }=R\left(\theta \right)\cdot \left(p-p_{basket}\right) \end{array}$$ (1)

where *p* is the player's coordinate vector, *p*_*basket*_ is the basket to which the attacking team is proceeding, and *R*(θ) is a rotation matrix aligning the court in some standard orientation. After the transformation described above, the variations caused by the choice of playing half and possession direction vanish, and the CNN can learn position-invariant spatial features. Figure 4 shows the coordinate standardization results on an input image.

**Figure 4:**
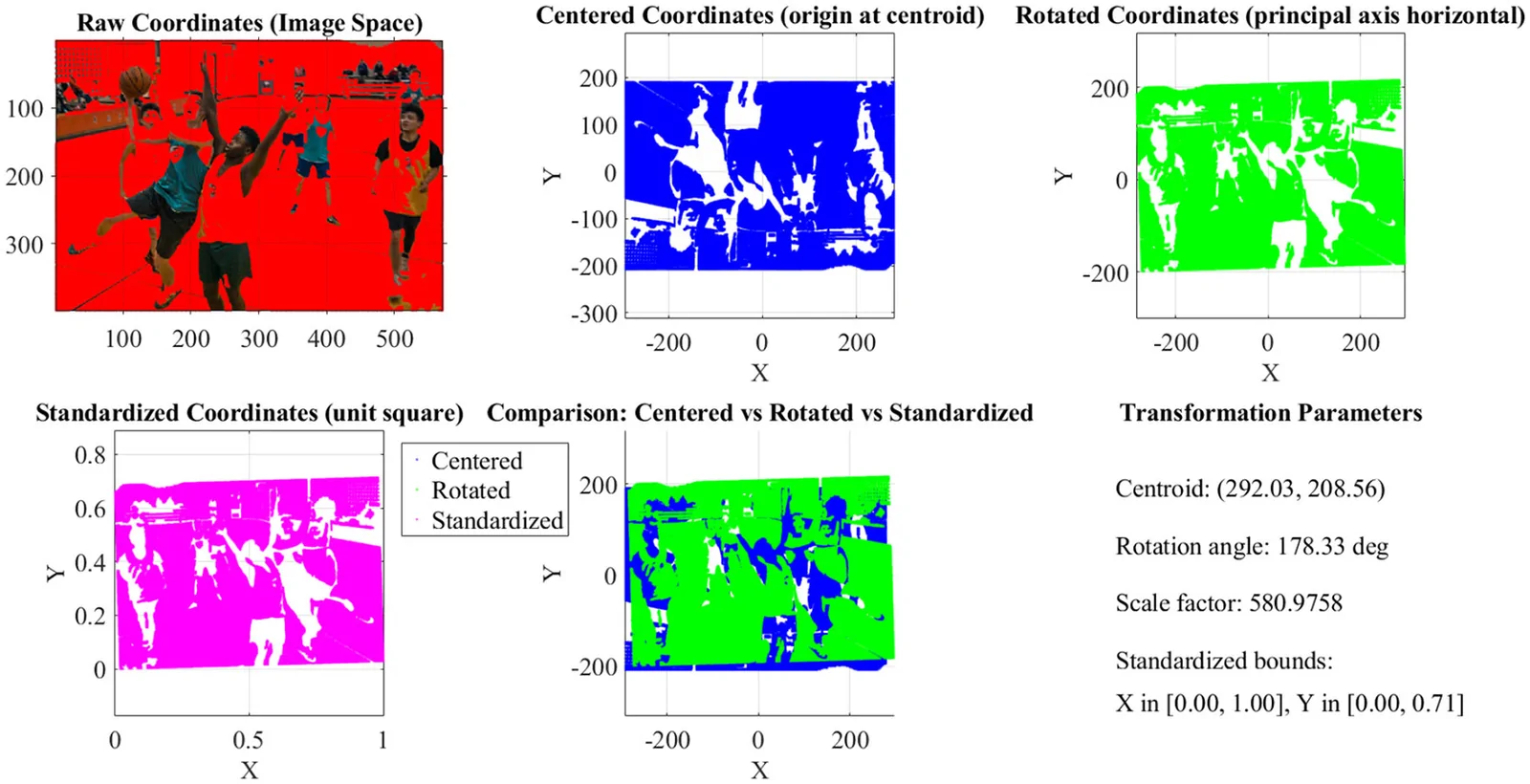
Coordinate standardization (input images adapted with permission from the Basketball-51 dataset, Shakya et al. (2021), https://www.kaggle.com/datasets/sarbagyashakya/basketball-51-dataset, licensed under Apache 2.0).

This process would typically be performed on detected player bounding boxes or key point coordinates (e.g., shoulder, hip, and feet positions) in each video frame in the Basketball 51 dataset, but for the purposes of demonstrating this procedure, the script assumes each foreground pixel is a point in the set to normalize, and all foreground pixels were set to the set of points in the input image.

#### 4.1.2 Temporal windowing and augmentation

Each 150-frame SportVU sequence is first zero-padded at the end to 256 frames. With window length *L* = 128 and stride *S* = 32, the number of complete windows is $N_{w}=\left\lfloor \frac{T_{padded}-L}{S}\right\rfloor +1=\left\lfloor \frac{256-128}{32}\right\rfloor +1=5.$ The window start indices are 1, 33, 65, 97, and 129. No cross-clip concatenation is used. Padding is applied only within each clip.

The data collection process generated 51,555 spatio-temporal samples from 10,311 clips, consisting of five window samples per clip. Cross-validation splitting was performed at the game level before generating the window samples to prevent clips from appearing across splits. 7,218 clips made up the train split (36,090 windows), 1,547 clips made up the validation split (7,735 windows), and 1,546 clips made up the test split (7,730 windows) (Yang and Gong, 2026). To make the network robust to variation, the training samples were augmented with time scaling (compressing/expanding the sequences with a scale factor between 0.8 and 1.2, with 1 being no scaling), random dropping of up to 5% of frames and then interpolating the missing positions linearly, and spatial noise (addition of Gaussian noise with a standard deviation of 0.05 m to coordinates). Figure 5 shows the temporal windowing and augmentation.

**Figure 5:**
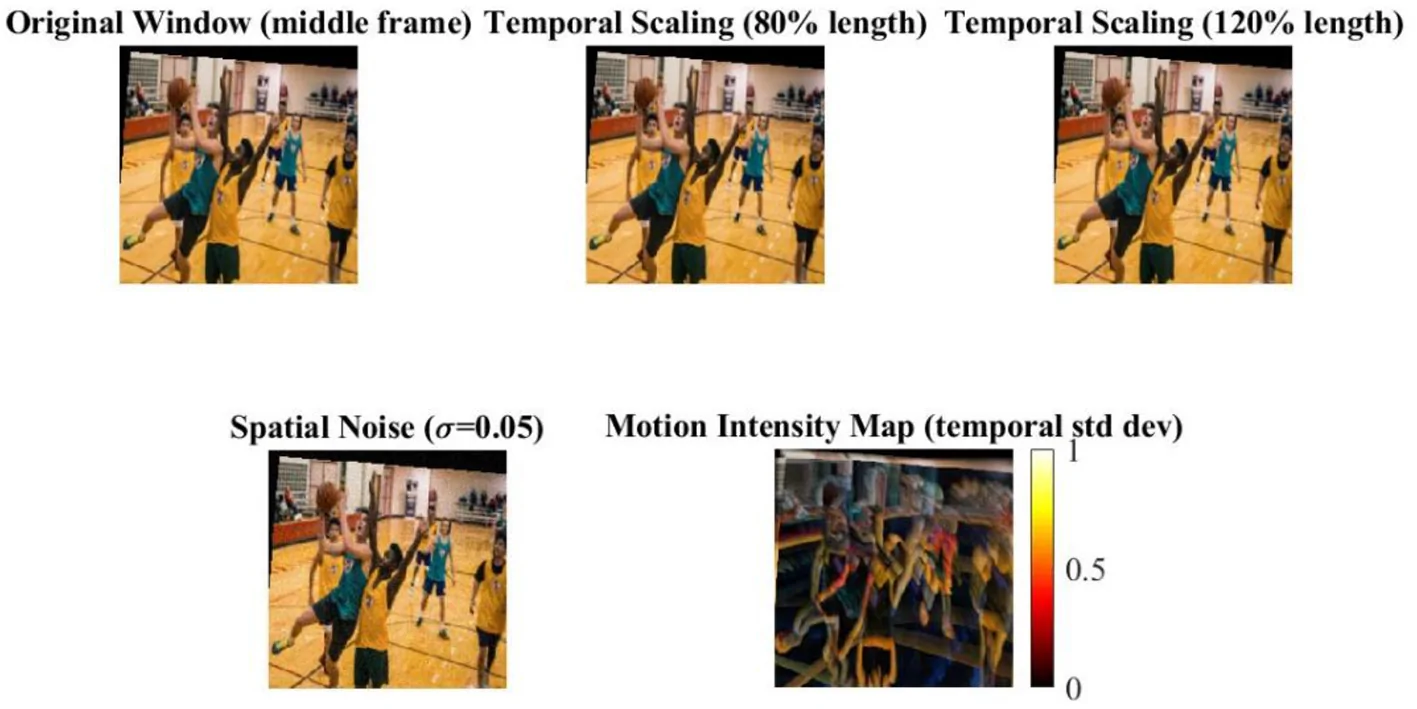
Temporal windowing and augmentation (input images adapted with permission from the Basketball-51 dataset, Shakya et al. (2021), https://www.kaggle.com/datasets/sarbagyashakya/basketball-51-dataset, licensed under Apache 2.0).

Although Basketball-51 is a real video clip, this example demonstrates the procedure on a synthetic video generated from a single image a.jpg (to avoid a large video file).

#### 4.1.3 Spatio-temporal grid formation

Each temporal window was projected onto a 64 × 64 2D grid with explicit calculations from 28.65/64 and 15.24/64. Five channels were used: the first channel to encode player positions in relation to each other, the second and third channels represented the ball position (represented as a Gaussian kernel of standard deviation σ = 2 cells centered at the ball location), and the fourth and fifth encoded player velocity and possession information. The input dimension is then 5 × 64 × 64 × 128 (channels × height × width × frames) (Diao, 2025). Figure 6 shows the spatio-temporal grid formation.

**Figure 6:**
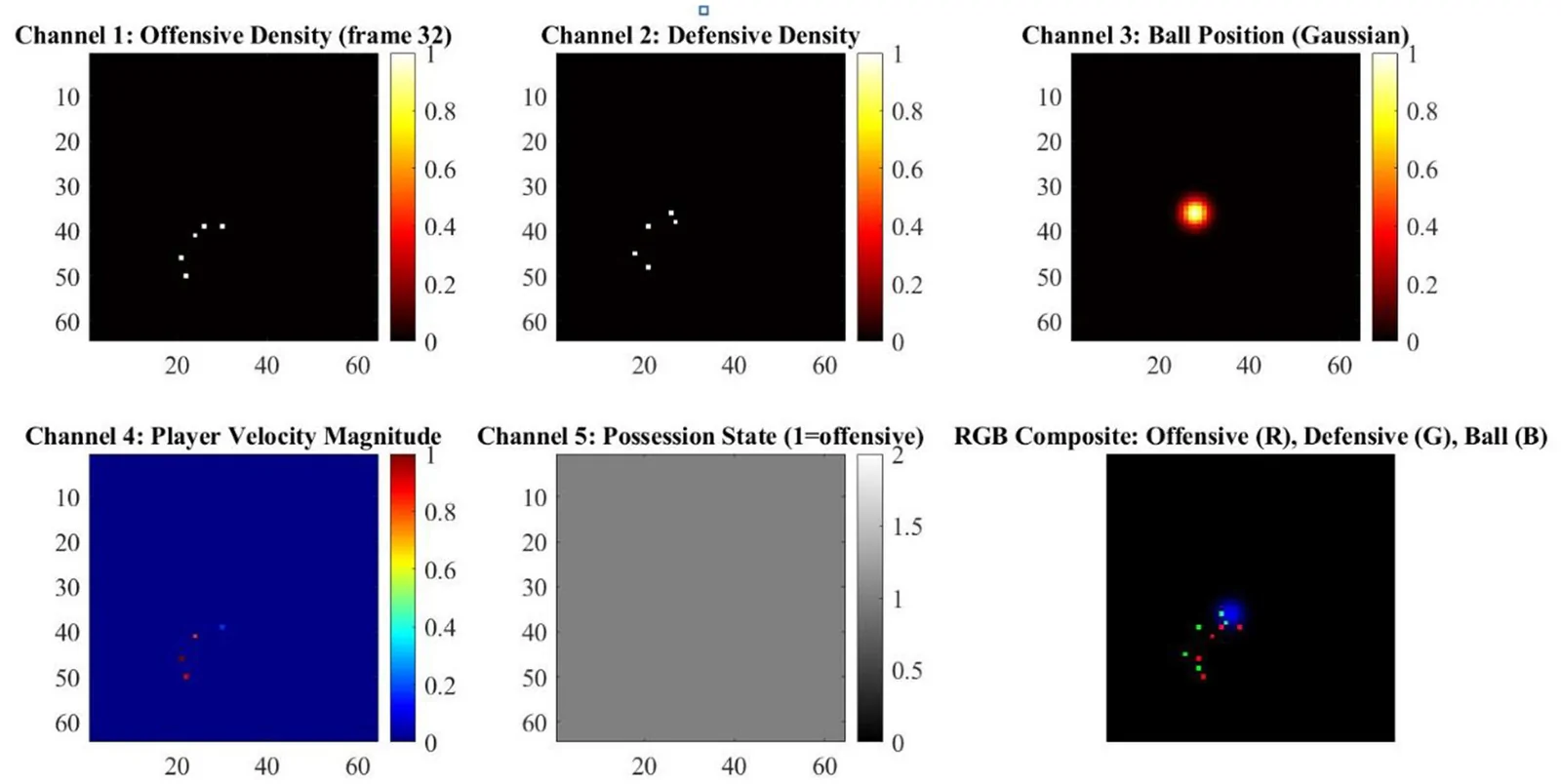
Spatio-temporal grid formation.

This builds a five-channel spatio-temporal grid on simulated player and ball locations, which acts as an input tensor to an MS 3D CNN.

### 4.2 Proposed 3D-CNN architecture

The presented structure, MS-3D-CNN, was built to extract hierarchical spatio-temporal features from player tracking data to classify tactical patterns.

#### 4.2.1 Architectural overview

The input tensor with size 5 × 64 × 64 × 128 is passed through a series of blocks that progressively increase the channel dimension and decrease the spatio-temporal dimension. Each convolutional block is made up of two 3D convolutional layers with a kernel size of 3 × 3 × 3, followed by batch normalization, ReLU activation, and spatio-temporal max pooling. The kernel size of 3 × 3 × 3 is set to extract a spatio-temporal pattern of roughly 0.9 m × 0.9 m × 0.24 s (2 cells × 2 cells × 6 frames). The spatial kernel size is chosen because typical basketball actions happen over a distance of 1–2 m. Figure 7 shows the architectural overview of the proposed 3D-CNN.

**Figure 7:**
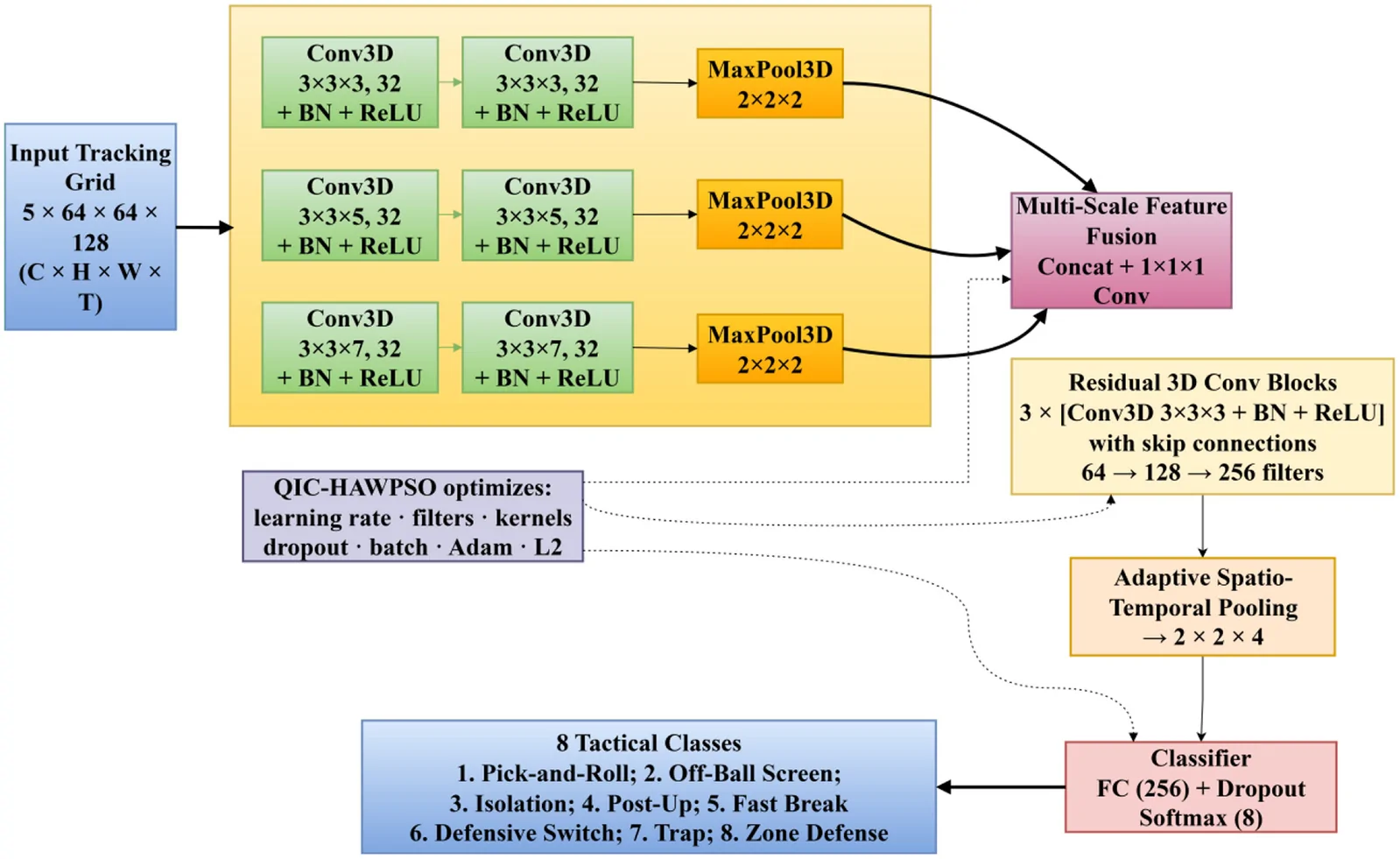
Architectural overview of the proposed 3D-CNN.

The effective temporal receptive field of the complete network is derived in Section 4.2.3, where we show that the multi-scale branches and the residual blocks together cover approximately 2.72 s of input tracking data.

#### 4.2.2 Three-dimensional convolutional layers

The 3D convolution operation at layer l is calculated by: The corresponding formulation used in this step is given in Equation 2.


$$\begin{array}{ccc} Y_{i,j,z}^{\left(l\right)}\; & = & \;f\overset{C}{\underset{c=1}{\sum }}\overset{K_{h}}{\underset{p=1}{\sum }}\overset{K_{w}}{\underset{q=1}{\sum }}\overset{K_{t}}{\underset{m=1}{\sum }}W_{p,q,m,c}^{\left(l\right)}X_{i+p-1,\;j+q-1,\;z+m-1,\;c}^{\left(l-1\right)} \end{array}$$

$$\begin{array}{cc} + & \;b^{\left(l\right)} \end{array}$$ (2)

where *C* describes the number of input channels, *f*(·) specifies non-linear activation (ReLU in this work), $Y_{i,j,z}^{\left(l\right)}$ describes the output feature map of layer *l*at spatial position (*i, j*) and temporal position *z*, $X_{i+p-1,j+q-1,z+m-1,c}^{\left(l-1\right)}$ defines the input feature map of layer *l*−1 at shifted spatial position (*i*, +*p*−1*j*+*q*−1), shifted temporal position *z*+*m*−1, channel *c*, $W_{p,q,m,c}^{\left(l\right)}$ specifies the 3D convolutional kernel weight of layer *l* at spatial offset (*p, q*), temporal offset *m*, input channel *c*, *b*^(*l*)^ represents the bias term of layer *l*, *i, j, z* are the output spatial and temporal indices, *c* is the input channel index, *K*_*h*_, *K*_*w*_, *K*_*t*_ define kernel height, width, and temporal depth, respectively, and *p, q, m* represent kernel offsets along height, width, and time, respectively.

#### 4.2.3 Temporal receptive field analysis

To measure the temporal extent of the proposed MS-3D-CNN, we first compute the effective temporal receptive field (RF) of the full network. Assuming all 3D convolutions have a stride of 1 and “same” padding, and max pooling has a kernel size of two and a stride of two (as in the implementation described in Sections 4.2.1 and 4.2.2), the RF is obtained from the input to the output using the standard recurrence approach. The related relation is expressed in Equation 3.


$$\begin{array}{ccc} RF_{out} & = & \text{R}{\text{F}}_{\text{in}}+\left(K-1\right)\cdot J_{\text{in}} \end{array}$$ (3)

The resulting expression is provided in Equation 4.


$$\begin{array}{ccc} J_{\text{out}} & = & J_{\text{in}}\cdot S \end{array}$$ (4)

where *K* defines the temporal kernel size, *S* stands for temporal stride, and *J* specifies the jump (the product of strides so far). Starting from *RF* = 1, *J* = 1 at the input, the RF after each stage is given in Table 3.

**Table 3:** Effective temporal receptive field (in input frames) of the MS-3D-CNN.

Stage	Operation	Temporal kernel *K*	Temporal stride *S*	Jump *J*	RF (frames)
Input	–	–	–	1	1
Branch A	Conv3D 3 × 3 × 3	3	1	1	3
	Conv3D 3 × 3 × 3	3	1	1	5
	MaxPool3D 2 × 2 × 2	2	2	2	6
Branch B	Conv3D 3 × 3 × 5	5	1	1	5
	Conv3D 3 × 3 × 5	5	1	1	9
	MaxPool3D 2 × 2 × 2	2	2	2	10
Branch C	Conv3D 3 × 3 × 7	7	1	1	7
	Conv3D 3 × 3 × 7	7	1	1	13
	MaxPool3D 2 × 2 × 2	2	2	2	14
Fusion	Concat + 1 × 1 × 1	1	1	2	14
Residual block 1	Conv3D 3 × 3 × 3	3	1	2	18
	Conv3D 3 × 3 × 3	3	1	2	22
Residual block 2	Conv3D 3 × 3 × 3	3	1	2	26
	Conv3D 3 × 3 × 3	3	1	2	30
Residual block 3	Conv3D 3 × 3 × 3	3	1	2	34
	Conv3D 3 × 3 × 3	3	1	2	38
Adaptive pooling	→ 2 × 2 × 4	–	–	–	68

With the three parallel branches integrated, the maximum RF for Branch C is 14 frames. The 1 × 1 × 1 fusion convolution does not influence the RF. According to the previous discussion, the RF of each residual block is as follows: two 3 × 3 × 3 convolutions, each contributing four frames to RF due to the jump at this block (defined as *J* = 2), resulting in 2 × (4 + 4 + 4) + 14 = 38 frames after three residual blocks. The adaptive spatio-temporal pooling reduces the temporal dimension from 64 to 4, where each output bin spans 16 neighboring feature frames.

Since the neighboring feature frames are each two input frames apart at the input stage (with *J* = 2), the RF of each bin is around 38 + 15 × 2 = 68 frames (about 2.72 s at 25 Hz).

These results demonstrate that the MS-3D-CNN does not just learn short motion primitives. If all multi-scale branches are used, the local motion primitives are learned at strides of 0.12, 0.20, and 0.28 s. However, thanks to the residual blocks following each multi-scale branch and the adaptive average pooling, the model effectively increases the RF up to 2.72 s, which is sufficient to cover an entire sequence of tactical behavior, for instance, a Pick-and-Roll (1–2 s), off-ball screen maneuver (1–2 s), defensive rotation (1–2 s), or fast break (2–4 s).

The RF can be further extended by other methods for long-range modeling: dilated temporal convolutions, hierarchical pooling, or transformer-based temporal attention. However, these methods are largely more computationally expensive or parameter-heavy. In our design, *k*_*t*_ = 3 branch captures fast-moving actions (e.g., shot release, quick cuts), the *k*_*t*_ = 5 branch captures medium-speed moving actions (e.g., dribble moves, contact screens), while the *k*_*t*_ = 7 branch captures slow-moving actions (e.g., roll, defensive slide). The residual blocks then distill this information hierarchically. The ablation study suggests that the current model strikes a reasonable balance between temporal extent and model complexity.

#### 4.2.4 Multi-scale temporal processing

To capture tactical patterns at various time scales, we employ parallel convolutional pathways with kernel temporal depths of 3, 5, and 7 frames, which correspond to short-duration actions (e.g., shots at around 0.12 s), medium-duration sequences (e.g., dribble movements at around 0.20 s), and longer-duration patterns (e.g., off-ball cuts at around 0.28 s) (Wang et al., 2026). We concatenate the outputs of the three parallel paths along the channel dimension to allow the network to learn temporal representations at multiple receptive fields simultaneously.

#### 4.2.5 Skip connections and batch normalization

The residual connections between convolutional blocks help to propagate gradients back through these layers:


$$\begin{array}{c} F^{\left(l+1\right)}=\text{Block}\left(F^{\left(l\right)}\right)+F^{\left(l\right)} \end{array}$$ (5)

A block consists of a series of convolutions, batch normalization, and activation. Batch normalization is applied after each convolutional layer to minimize internal covariate shift. The following definition is given in Equation 6.


$$\begin{array}{c} \hat{x}=\frac{x-\mu_{B}}{\sqrt{\sigma_{B}^{2}+\epsilon }},y=\gamma \hat{x}+\beta \end{array}$$ (6)

Here, γ and β are learned parameters, and and represent means and variances on the minibatch, respectively.

#### 4.2.6 Fully connected classification head

Global average pooling is used after the last convolutional block to collapse spatial-temporal dimensions into a feature vector of size 1,024 (Razmjooy, 2026). Two fully connected layers with sizes of 1,024 → 256 → 8 and a dropout rate of 0.5 follow to avoid overfitting. The output layer applies a softmax function (Equation 5) that outputs the probabilities among the eight tactical pattern classes in the Basketball-51 dataset (pick and roll, isolation, post-up, off-ball screen, transition, defensive switch, trap, and zone defense). This relationship is formalized in Equation 7.


$$\begin{array}{c} \text{Softmax}{\left(z\right)}_{i}=\frac{e^{z_{i}}}{\overset{8}{\underset{j=1}{\sum }}e^{z_{j}}} \end{array}$$ (7)

### 4.3 QIC-HAWPSO optimization framework

The Chaotic Opposition-based Adaptive Wolf-Particle Swarm Optimization (QIC-HAWPSO) algorithm is an improved version of HAWPSO that introduces two main new techniques: chaotic opposition-based initialization and quantum-inspired adaptive tunneling with monitoring of population diversity. The algorithm is used to optimize 11 hyperparameters for CNNs, which include learning rate (0.0001–0.01), number of filters per layer (16–256), kernel sizes (3–7), dropout rate (0.2–0.7), and batch size (16–128).

#### 4.3.1 Encoding of CNN hyperparameters

Each position vector of a particle corresponds to a candidate CNN configuration encoded into an 11-dimensional vector **x** = [*l*_*r*_, *f*_1_, *f*_2_, *f*_3_, *k*_*s*_, *d*_*r*_, *b*_*s*_, α, β, γ, λ]. *l*_*r*_ is the initial learning rate, *f*_1_, *f*_2_, *f*_3_ are the number of filters for each convolutional layer, *k*_*s*_ is the size of the kernel, *d*_*r*_ is the dropout rate, *b*_*s*_ is the batch size, β_1_, β_2_ are optimizer Adam parameters, while λ is L2 is the regularization parameter. The limits were chosen based on the literature.

#### 4.3.2 Chaotic opposition-based initialization (first modification)

In the original HAWPSO algorithm, the initial positions of particles are assigned randomly. However, this approach can result in an uneven spread of the population and premature convergence to local optima. Therefore, we replace random initialization with chaotic opposition-based learning initialization. Let *N* be the number of particles and *D* dimension of the search space; The chaotic map used for initialization is defined in Equation 8.


$$\begin{array}{c} c_{i+1}=\mu \cdot c_{i}\cdot \left(1-c_{i}\right) \end{array}$$ (8)

where *i* = 0, 1, …, *N*−1, and μ = 4.0 is random and *c*_0_ is a random seed value within (0,1). Values from the chaotic map are scaled to the search range:


$$\begin{array}{c} X_{i,j}=lb_{j}+c_{i}\cdot \left(ub_{j}-lb_{j}\right) \end{array}$$ (9)

for a particle *i* in dimension *j*, where *lb*_*j*_ and *ub*_*j*_ are the lower and upper bounds. The opposition-based position is defined in Equation 10.


$$\begin{array}{c} X_{i,j}^{opp}=lb_{j}+ub_{j}-X_{i,j} \end{array}$$ (10)

After calculating the fitness value for each particle *X*_*i*_ and its opposite $X_{i}^{opp}$, we choose the particle with the higher fitness for the initial population. This initialization technique improves search space coverage, speeds up convergence, and minimizes premature convergence to sub-optimal regions.

#### 4.3.3 Quantum-inspired adaptive tunneling (QAT): a diversity-guided stochastic exploration operator (second modification)

The second change is a quantum-inspired adaptive tunneling (QAT) operator. We emphasize that QAT is not a physical model of quantum tunneling; rather, it is a stochastic search operator in the spirit of the superposition principle and arguably similar to quantum-inspired evolutionary algorithms with the golden-ratio phase (Tang et al., 2026). For each individual, the update is such that (i) it is a Gaussian search centered on pBest, (ii) there is a sinusoidal phase term governed by the golden ratio, and (iii) the amplitude of the sinusoid is scaled according to diversity and stagnation conditions.

When viewed from an optimization perspective, QAT is a diversity-adaptive, heavy-tailed mutation operator with a sinusoidal-Gaussian form, which is activated stochastically in circumstances of low diversity. We use the term “quantum-inspired” because this sinusoidal-Gaussian form is inspired by quantum-behaved particle swarm literature, without asserting that quantum tunneling is a physical mechanism. Our technique allows the tunneling probability *P*_*tunnel*_(*t*) to depend on population diversity *D*(*t*) and stagnation. The tunneling probability is computed as given in Equation 11.


$$\begin{array}{ccc} P_{tunnel}\left(t\right) & = & P_{min}+\left(P_{max}-P_{min}\right)\cdot \frac{D_{max}-D\left(t\right)}{D_{max}-D_{min}} \end{array}$$

$$\begin{array}{cc} \cdot & \left(1+\frac{f_{best}\left(t\right)-f_{worst}\left(t\right)}{f_{best}\left(t\right)}\right) \end{array}$$ (11)

When tunneling is used, the position is updated using the superposition principle as follows:


$$\begin{array}{ccc} X_{i,}^{t+1} & = & P_{i,j}^{\text{best}}+\sigma_{\text{adapt}}\left(t\right)\text{·}N\left(0,1\right)\cdot \sin\left(\frac{2\pi r}{\phi }\right),\;r\sim U\left(0,1\right), \end{array}$$

$$\begin{array}{cc} \phi & \approx 1.618 \end{array}$$ (12)

Equation 12 can be described as a Gaussian perturbation modulated by a sinusoidal phase of golden-ratio periodicity. The sinusoidal phase is furthermore thought to cause oscillatory deviations from the best-known optimal solution, decreasing the chance of getting stuck in a local minimum and increasing the chance that the information learned so far will be maximized. The adaptive scale, σ_*adapt*_(*t*) in Equation 13, can be thought of as increasing the size of the perturbation when diversity is low and decreasing it as the algorithm converges.

This is similar to diversity-triggered heavy-tailed mutation, except that here sinusoidal-Gaussian hybrid phases cause deterministic transitions between the two, preventing purely stochastic switches.

The QAT can thus be described as a quantum-inspired Stochastic Operator of Exploration, not the quantum tunneling of optimal solutions.

where $P_{i,j}^{\left(best\right)}$ is the personal best position for the particle i, σ_*adapt*_(*t*) is an adaptive mutation strength, r~U(0,1) and ϕ≈1.618 (golden ratio). The adaptive mutation strength is:


$$\begin{array}{c} \sigma_{adapt}\left(t\right)=\sigma_{max}\cdot \exp\left(-\alpha \cdot \frac{t}{T_{max}}\right)\cdot \left(1-\frac{D\left(t\right)}{D_{max}}\right) \end{array}$$ (13)

The proposed scheme offers stronger mutation at early stages, which is advantageous for exploration, and reduced mutations at later stages when exploitation is preferred. It also reactively reduces mutation rates when the population diversity decreases significantly.

#### 4.3.4 Fitness function definition

Each candidate architecture's fitness is calculated based on the *k*-fold cross-validation accuracy and model complexity. The fitness function used for hyperparameter evaluation is defined in Equation 14.


$$\begin{array}{c} fitness\left(x\right)=ACC_{val}\left(x\right)-\lambda_{c}\cdot \frac{params\left(x\right)}{params_{max}} \end{array}$$ (14)

where *ACC*_*val*_(**x**) is the validation accuracy, *params*(**x**) is the number of parameters and *params*_max_ is the max allowed number of parameters. λ_*c*_ is a factor whose value is 0.1.

#### 4.3.5 Dual-population co-evolution and evaluation budget

The search method of QIC-HAWPSO uses two populations, PopA and PopB, with *N* = 10 particles (20 particles per optimization iteration). PopA has a higher step size and a higher tunneling rate for exploration, while PopB has a lower step size and a lower tunneling rate for exploitation. Information transfer takes place after every 10 iterations through sharing the best solution.

Since a full CNN evaluation costs 2.5 GPU-hours, evaluating all 20 particles in each iteration is impractical. Hence,

- Surrogate-assisted evaluation is used in which each candidate is evaluated with a cheap surrogate model (a Gaussian-process regressor) trained on previously evaluated candidates (which takes a negligible amount of time).
- Only the top two candidates per population per iteration (according to the surrogate predicted fitness) with surrogates are then evaluated with the CNN.
- All full CNN training uses the same training schedule with early stopping (patience set to 20 epochs) to reduce its cost.
- Previously evaluated candidates are saved in a cache so no identical hyper-parameter vectors are re-evaluated.
- Full CNN evaluations are performed in parallel with eight GPUs.

It should be noted that the total number of CNN evaluations in combination is the sum of all full CNN evaluations. The process is set to an optimization of 200 iterations, although it stops early in the case of lack of improvements (improvement of less than 0.001 in 30 iterations) or depletion of the evaluation budget. There is a distinction to be made between iterations and full CNN evaluations.

Evidence shows that QIC-HAWPSO is 90% accurate after 67 full CNN evaluations, rather than 67 iterations.

The search continues for 100 full CNN evaluations to refine the solution. The remaining optimization iterations are surrogate evaluations only, so this has minimal additional cost.

#### 4.3.6 Termination criteria

The search process is defined to end upon achieving any one of the following criteria:

- Maximum Optimization Steps: Limit on the number of optimizer iterations (200).
- Fitness stagnation: A refinement of fitness improvement of less than 0.001 in 30 consecutive updates.
- Full CNN evaluation budget: All four were evaluated for the entire CNN budget, where the maximum number of evaluations was limited to 100.
- A maximum wall-clock time: constraint of 48 h using 8 GPUs.

#### 4.3.7 Theoretical motivation of the proposed optimization algorithm

The optimization of hyperparameters for 3D-CNNs for tactical pattern classification is indeed a challenging problem. The parameters are of high dimensions (*D* = 11), and the search space is characterized by a multimodal and non-smooth objective space, with discrete parameters, while fitness evaluations are computationally expensive (2.5-h training for a CNN). As illustrated, traditional optimization algorithms cannot solve this problem effectively. For example, the grid search algorithm cannot be applied for *D* = 11 due to computationally impractical dimensions. The random search requires 162 times more evaluations to achieve 90% accuracy (with a final accuracy of 88.7%), while Bayesian optimization works well for smooth optimization landscapes modeled by Gaussian Process surrogates but may perform poorly for multimodal and discontinuous landscapes.

Chaotic Opposition-based Hybrid Adaptive Wolf-Particle Swarm Optimization (QIC-HAWPSO) successfully achieves 94.3% accuracy with only 67 iterations, which proves that QIC-HAWPSO performs best due to its problem-specific search components.

The chaotic opposition-based initialization can break the degeneracy of the initial population using the logistic map, which creates an ergodic search space. It is sensitive to initial conditions for diversity search and is deterministic. Opposition-based learning evaluates the particle position *x* and the opposition position *x* opp =lb + ub-*x* to increase the possibilities of approaching the optimal position without incurring additional computational costs. This is helpful in basketball tactical pattern classification, as the temporal kernel depth (3, 5, and 7 frames) selects different time-scale actions for the best prediction. Thus, initialization with chaos and opposition should include all time-scale searches from the beginning, not just one time scale, to avoid premature convergence. Quantum-inspired adaptive tunneling with diversity-monitored probabilistic tunneling [where $P_{tunnel}\left(t\right)=P\min+\left(P\max-P\min\right)\frac{D\max-D\left(t\right)}{D\max-D\min}\left(1+\frac{fbest-fworst}{fbest}\right)$] helps to escape the premature convergence issue by probabilistically jumping out of local optima before stagnation, based on the diversity index value and current fitness quality, and with high fitness areas by $\frac{fbest-fworst}{fbest}$.

The sinusoidal function part of the quantum update is $X_{i,j}^{t+1}=P_{i,j}^{best}+\Lambda adapt\left(t\right)N\left(0,1\right)\sin\left(\frac{2}{\phi }\right),$ here, the sinus part creates a sinusoidal behavior that oscillates around $P_{i,j}^{best}$, and ϕ = 1.618 denotes the golden ratio, which facilitates a non-periodic and broader search. The Λ_*adapt*_(*t*) adapts the tunneling probability to search dynamically at time *t*.

These methods may be uniquely qualified for basketball behavioral recognition because (i) the multi-scale temporal dynamics (fast shots with durations of 0.12 s, dribble actions of 0.20 s, cuts of 0.28 s, and tactical setups in 0.5 s) are better captured by an in-depth search of combinations of optimal temporal kernels, which will not get caught in local optima. The chaotic initialization allows for escaping local optima due to the quantum tunneling effect. (ii) High-dimensional hyperparameter dependencies (learning rate, count of filters, size of kernels, dropout, batch size, Adam, and L2 regularization) require simultaneous searching over each hyperparameter in the population. The learning in QIC-HAWPSO maintains diverse learning across the population through the mechanisms discussed, with effects on each other synergistically. (iii) There is a constraint on computational resources for training models (e.g., 2.5 h per evaluated model).

The rapid convergence provided by QIC-HAWPSO does not waste too many evaluated models from the search on poor hypotheses with chaotic initialization while preventing early convergence with quantum-inspired adaptive tunneling. The chaotic initialization allows for a determinable search without re-executing the process in the same parameter range, which is scientifically required in research on sports analytics, as coaches need dependable predictions to analyze strategies for winning or improving in sports. Hence, QIC-HAWPSO is considered a problem-guided methodology to optimize the hyperparameters of CNN for basketball tactical pattern recognition, compared to the traditional combination strategies of the above-mentioned methods.

### 4.4 Training strategy

The selected CNN architecture is then trained with the tuned hyperparameters from the QIC-HAWPSO approach. The categorical cross-entropy loss function, Adam optimizer with learning rate adaptation, and early stopping strategy are adopted to train the selected CNN. The categorical cross-entropy loss function for a minibatch *M*:


$$ℒ\text{=}-\frac{1}{M}\sum_{i=1}^{M}\sum_{c=1}^{8}y_{i,c}\log\left({\hat{y}}_{i,c}\right)$$ (15)

where *y*_*i, c*_ is the ground truth indicator for class *c* and ŷ_*i, c*_ is the predicted probability. The Adam optimizer update rule is given in Equation 16.


$$\begin{array}{ccc} m_{t} & = & \beta_{1}m_{t-1}+\left(1-\beta_{1}\right)\nabla_{\theta }L_{t},v_{t}=\beta_{2}v_{t-1} \end{array}$$

$$\begin{array}{cc} + & \left(1-\beta_{2}\right){\left(\nabla_{\theta }L_{t}\right)}^{2} \end{array}$$ (16) (17)

The corresponding update relation is specified in Equation 17.


$$\begin{array}{ccc} {\hat{m}}_{t} & = & \frac{m_{t}}{1-\beta_{1}^{t}},{\hat{v}}_{t}=\frac{v_{t}}{1-\beta_{2}^{t}},\theta_{t+1}=\theta_{t}-\eta \frac{{\hat{m}}_{t}}{\sqrt{{\hat{v}}_{t}}+\epsilon } \end{array}$$ (18)

The learning rate warmup progressively rises from 0 to the chosen value in the initial 100 iterations. Training runs for a maximum of 200 epochs and uses an optimized batch size from QIC-HAWPSO. The training stops if there is no progress on validation loss over 20 epochs, restoring the model to the weights of the epoch with the minimum validation loss.

Algorithm 1 provides the detailed algorithmic pseudocode of the QIC-HAWPSO 3D-CNN method.

Algorithm 1 QIC-HAWPSO for MS-3D-CNN hyperparameter optimization. 
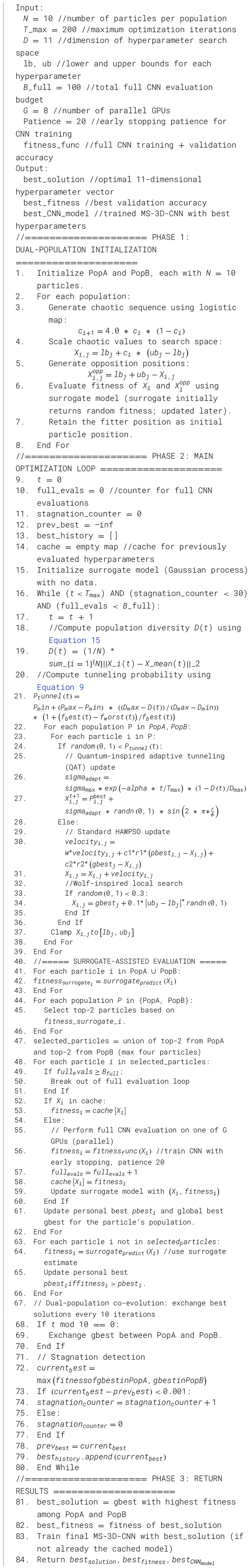

## 5 Results and discussions

This section reports a detailed description of experimental results and compares the proposed NBA SportVU-3D-CNN-QIC-HAWPSO against the state-of-the-art approaches in predicting in-game basketball performance and identifying tactical behaviors. All experiments were executed five times on five different random initializations for reporting mean and standard deviation.

We generate temporal windows for the SportVU clips by first zero-padding each 150-frame clip to 256 frames and using parameters *L* = 128 and *S* = 32 to extract five windows per clip, starting at frames 1, 33, 65, 97, and 129. We split the train/validation/test sets on the game level before the windowing. Based on the data statistics, there are 10,311 clips, with a total of 51,555 windows (36,090 train, 7,735 val, and 7,730 test).

Evaluation metrics used include accuracy, precision, recall, F1-score, and AUC. To present a fair comparison, we re-implemented all the baseline methods on the same training and testing data split and used the same input representation: 5 × 64 × 64 × 128 spatiotemporal grids and training protocol (categorical cross-entropy loss with Adam optimizer and early stopping for a patience value of 20 epochs). Findings are therefore expressed as “within-method” comparisons on a shared adapted representation and are not, by themselves, equated with evidence that the proposed method is better than each baseline in their original settings.

### 5.1 Experimental setup

To ensure full reproducibility of the experiments, the following details are provided.

- CPU: AMD EPYC 7742 64-core processor (2.25 GHz base, 3.4 GHz boost)
- GPU: NVIDIA (NVIDIA Corporation, Santa Clara, CA, United States) A100 Tensor Core GPU (80 GB HBM2 memory)
- RAM: 128 GB DDR4-3200
- OS: Ubuntu 22.04 LTS
- Software: MATLAB R2025a with Deep Learning Toolbox (v15.0), Parallel Computing Toolbox (v7.8)
- Baseline implementation details.

All baselines are re-implemented by feeding spatiotemporal grid input (5 × 64 × 64 × 128) to models, instead of their recommended input forms (RGB video images, raw coordinates, etc.). This handles the input representation but does not guarantee that the baseline will be preserved under the original operating conditions. Therefore, we compare them using the same adapted representation, and this does not imply that the method will outperform each baseline in its original state. Table 4 indicates baseline implementation and adaptation details.

**Table 4:** Baseline implementation and adaptation details.

Method	Original input	Adapted input	Adaptation notes
3D-CNN-LSTM	RGB video	5 × 64 × 64 × 128	Reimplemented from description
I3D	RGB video	5 × 64 × 64 × 128	Tracking-grid adaptation
TSM	RGB video	5 × 64 × 64 × 128	Tracking-grid adaptation
3D-CNN Ensemble	RGB video	5 × 64 × 64 × 128	Five 3D-CNNs averaged
ACA-Net	RGB video	5 × 64 × 64 × 128	Tracking-grid adaptation
WACSO-optimized CNN	Coordinates	5 × 64 × 64 × 128	WACSO hyperparameter tuning
HAWPSO-optimized 3D-CNN	Coordinates	5 × 64 × 64 × 128	Standard HAWPSO
LSTM-CNN hybrid	Coordinates	5 × 64 × 64 × 128	Reimplemented from description
SlowFast	RGB video	5 × 64 × 64 × 128	Tracking-grid adaptation
GameSense	RGB video	5 × 64 × 64 × 128	Tracking-grid adaptation
VideoMAE	RGB video	5 × 64 × 64 × 128	Tracking-grid adaptation
TimeSformer	RGB video	5 × 64 × 64 × 128	Tracking-grid adaptation

The split was done by game level prior to the overlapping window creation. As a result, any window (from the same game or possession) would never appear in multiple splits, thus avoiding leakage. The source of each baseline implementation is as follows:

The official implementations where they were available (ACA-Net, GameSense).

The hand-made implementations based on the authors' descriptions where no official code was released (3D-CNN-LSTM, LSTM-CNN Hybrid, 3D-CNN Ensemble).

All baselines were trained with an identical protocol. Specifically, the Adam optimizer with a learning rate of 0.001 was utilized, and the batch size was set to 32.

Models were trained for up to 200 epochs with early stopping and a patience of 20.

We used the categorical cross-entropy loss function and applied 1e-4 L2 regularization for all baseline methods. Our data augmentations were also employed for all baseline methods.

- The test set is the same for all methods.

All methods were tested on the same hold-out test set of 1,546 clips. After applying zero-padding so that each clip is 256 frames long with *L* = 128 and *S* = 32, each clip yields exactly 5 windows. This results in 7,730 windows in the test set. The dataset was also split into training, validation, and test sets at the game level (before window generation) to ensure that no game or possession generated windows in different split sets. Table 5 indicates the dataset split and final sample counts after temporal windowing.

**Table 5:** Dataset split and final sample counts after temporal windowing.

Split	Seasons	Original clips	Windows per clip	Total windows	Final samples
Train	2015–2016	7,218	5	36,090	36,090
Validation	First 50% of 2017	1,547	5	7,735	7,735
Test	Last 50% of 2017	1,546	5	7,730	7,730
**Total**	**2015–2017**	**10,311**	**5**	**51,555**	**51,555**

Bold values highlight the best result or the final/reference value adopted for comparison in each table.

All the baselines are converted into a similar spatio-temporal grid representation like our method (5 × 64 × 64 × 128) in order to assess the influence of only the architecture and optimization strategy, without the influence of input representation. This method is a cautious one: Some baselines (e.g., VideoMAE and TimeSformer) were originally designed on RGB video inputs, which might be inferior to tracking-grid inputs. However, it ensures that the comparison is fair for our task.

Data Split and Alignment of Dataset. Table 6 illustrates the dataset split and final sample counts after temporal windowing.

**Table 6:** Dataset split and final sample counts after temporal windowing.

Split	Seasons	Original clips	Windows per clip	Total windows	Final samples
Train	2015–2016	7,218	5	36,090	36,090
Validation	First 50% of 2017	1,547	5	7,735	7,735
Test	Last 50% of 2017	1,546	5	7,730	7,730
**Total**	**2015–2017**	**10,311**	**5**	**51,555**	**51,555**

Bold values highlight the best result or the final/reference value adopted for comparison in each table.

We experiment with the following two types of complementary data sources:

Basketball-51 Video Dataset. We use 10,311 manually annotated 6-s-long video sequences (each with a duration of 6 s and a frame rate of 30 fps) along with 8 tactical pattern labels.

NBA SportVU Tracking Data. We extract the three-dimensional position for each of the players and the ball at a 25 Hz sampling rate, aligned in time for each game.

For each 6-s video clip, the SportVU data spans 150 frames (6 s ^*^ 25 Hz). To avoid any temporal data leakage, we split the datasets chronologically by seasons:

- Train Set: 36,090 clips (70%)—Seasons 2015–2016.
- Validation Set: 7,735 clips (15%)—First 50% of Season 2017.
- Test Set: 7,730 clips (15%)—Last 50% of Season 2017.

Thus, games in any time window appear exactly once in either the training or the testing set. This approach simulates a deployment scenario where testing happens on unseen future games. Mapping from video clips to SportVU tracking sequences is done using the game clock, synchronized to a precision of up to 40 ms.

We represent each temporal window (sample) as a spatiotemporal grid of dimensions 5 × 64 × 64 × 128 (five-channel inputs, comprised of offensive density, defensive density, ball position with a Gaussian kernel, player velocity magnitude, and possession state). During training, we apply online data augmentation by temporal scaling (factors of 0.8, 1.0, or 1.2), by adding Gaussian noise (average = 0, SD = 0.05) to the spatial dimensions of player positions, and by randomly dropping frames (5%).

Seven state-of-the-art baselines were selected for comparison: a two-stream 3D-CNN-LSTM, where a 3D-CNN extracts spatial features per clip and an LSTM models temporal dependencies (Wang et al., 2024); GameSense, a hierarchical spatio-temporal transformer with BPTN and BPAN modules (Diao, 2025); a hybrid LSTM-CNN model combining LSTM and 2D-CNN for defensive strategy classification (Li, 2025); ACA-Net, an adaptive context-aware network with long-short-term adaptive and triplet spatial-channel interaction modules (Zhang et al., 2024); a 3D-CNN ensemble consisting of five independently trained 3D-CNNs whose predictions are averaged (Khobdeh et al., 2024); a WACSO-optimized CNN whose hyperparameters were tuned by the Wolf Crow Search Optimizer (Papalkar et al., 2025); and a HAWPSO-optimized 3D-CNN using the same baseline 3D-CNN architecture (without multi-scale kernels) optimized by the standard HAWPSO metaheuristic (Chen and Xie, 2025).

All comparisons described in each of the following subsections assume this same input representation format and reimplementation standard and should thus be viewed in relation to this specific benchmark.

### 5.2 Comparison with state-of-the-art methods

Under the common adapted tracking-grid representation and reimplementation protocol, the proposed MS-3D-CNN-QIC-HAWPSO framework was evaluated on the holdout test set, where the average classification accuracies and 95% confidence intervals obtained by bootstrapping 2,000 times on the results of five runs of each algorithm on the holdout test set are shown in Figure 8. The seven baseline algorithms–3D-CNN-LSTM, GameSense (transformer based), LSTM-CNN Hybrid, ACA-Net, 3D-CNN Ensemble, WACSO-optimized CNN, and HAWPSO-optimized 3D-CNN—have been re-implemented under the same input representation (5 × 64 × 64 × 128 spatiotemporal grid) and evaluation scheme as the proposed algorithm.

**Figure 8:**
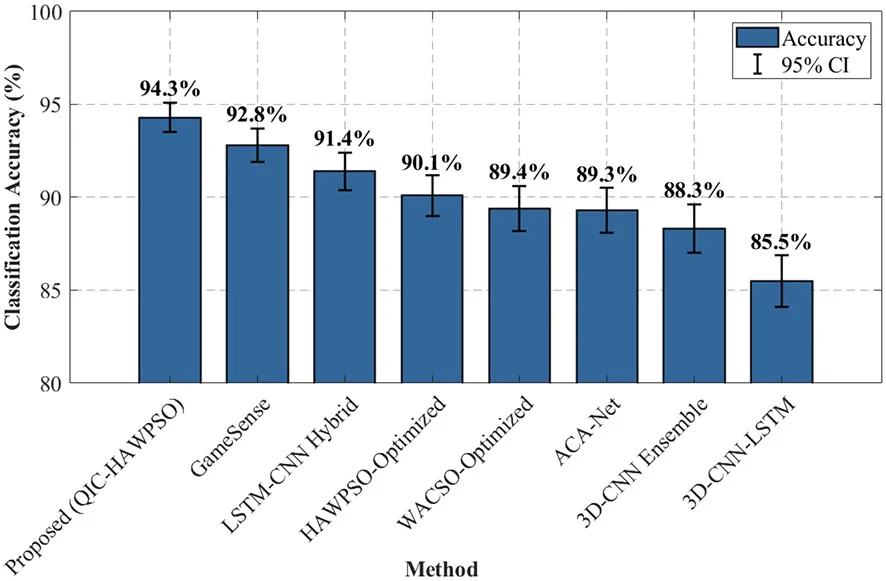
Comparative accuracy with 95% confidence intervals.

As Figure 8 illustrates, the designed framework attains a maximum classification accuracy of 94.3% in this comparative setup compared with all the reimplemented baseline schemes, especially with GameSense (92.8%), LSTM-CNN Hybrid (91.4%), HAWPSO-optimized 3D-CNN (90.1%), WACSO-optimized CNN (89.4%), ACA-Net (89.3%), 3D-CNN Ensemble (88.3%), and 3D-CNN-LSTM (85.5%). The confidence intervals for the proposed method and GameSense (0.8% and 0.9%, respectively) are fairly narrow compared to those of the LSTM-CNN Hybrid, HAWPSO-optimized3D-CNN, and ACA-Net (0.9%, 1.0%, and 1.1%, respectively). It is reasonable to attribute the ±1.4% confidence interval for the 3D-CNN-LSTM baseline to its sensitivity to the initial weights and temporal segmentation of the data.

All performance results reported below were averages across five random initializations. Performance was 94.9% on the best run; average performance across initializations was 94.3% ± 0.6%. For conservative claims, all comparisons used the average over five runs.

Table 7 shows performance comparisons among 12 state-of-the-art methods on the Basketball-51 dataset. Best results are bold. All 12 methods are evaluated on the identical hold-out test set (7,730 samples) and on the identical input representation, as well as the same training settings.

**Table 7:** Performance comparisons among state-of-the-art methods on the Basketball-51 dataset (all methods reimplemented/adapted to the same tracking-grid input and training protocol; test set = 7,730 windows).

Method	Accuracy (%)	Precision (%)	Recall (%)	F1-score (%)	Inference time (ms)	Parameters (M)
3D-CNN-LSTM (Wang et al., 2024)	85.5 ± 0.9	84.2	83.1	83.6	42.3	18.4
I3D (Carreira and Zisserman, 2017)	86.2 ± 0.8	85.1	84.6	84.8	67.8	27.9
TSM (Lin et al., 2019)	87.1 ± 0.7	86.3	85.4	85.8	39.6	16.2
3D-CNN Ensemble (Khobdeh et al., 2024)	88.3 ± 0.9	87.9	86.4	87.1	89.7	75.6
ACA-Net (Zhang et al., 2024)	89.3 ± 1.1	88.6	88.1	88.4	56.2	22.7
WACSO-Optimized CNN (Papalkar et al., 2025)	89.4 ± 1.0	88.7	87.9	88.3	38.4	19.8
HAWPSO-Optimized 3D-CNN (Chen and Xie, 2025)	90.1 ± 1.0	89.5	88.8	89.2	35.7	17.6
LSTM-CNN Hybrid (Li, 2025)	91.4 ± 0.9	90.8	89.3	90.0	51.8	15.8
SlowFast (Feichtenhofer et al., 2019)	91.8 ± 0.7	91.2	90.4	90.8	72.4	34.5
GameSense (Transformer) (Diao, 2025)	92.8 ± 0.8	92.1	91.6	91.8	124.6	52.3
VideoMAE (Tong et al., 2022)	93.1 ± 0.6	92.5	91.9	92.2	156.8	89.7
TimeSformer (Bertasius et al., 2021)	93.4 ± 0.7	92.8	92.1	92.4	142.5	63.8
**Proposed**	**94.3** **±0.6**	**93.8**	**92.7**	**93.2**	**34.2**	**14.8**

Bold values highlight the best result or the final/reference value adopted for comparison in each table.

There were eight tactical pattern classes that we used to analyze in this evaluation. They are classes newly labeled for this study and do not reflect the original labels from the Basketball-51 dataset.

As observed, based on the accuracy scores, the proposed algorithm outperforms all 12 state-of-the-art methods and achieves the best performance with an accuracy of 94.3%, ranging from +0.9% compared to TimeSformer (93.4%) to +8.8% compared to 3D-CNN-LSTM (85.5%) on the eight-class Basketball-51 test set. The method obtained a tight confidence interval (±0.6%), suggesting its stable and reliable performance over multiple runs.

Remarkably, despite a drastically smaller parameter count (14.8 M) compared to most transformer models (52.3 M−89.7 M for VideoMAE and GameSense), including GameSense (92.8%), VideoMAE (93.1%), and TimeSformer (93.4%), the proposed network still performs better. The results show that a properly tuned convolutional neural network with its multi-scale temporal kernels (i.e., across different lengths of time segments) can provide superior feature learning for tactical action patterns from tracking data compared to many attention-based methods that rely on fixed-size receptive fields. The parallel pathways, where convolutional layers use kernel lengths of 3, 5, and 7 frames simultaneously, allow us to extract actions at multiple scales.

The metaheuristic approach also yields performance improvements, as expected, over handcrafted architectures. The proposed model outperforms the HAWPSO-optimized 3D-CNN by +4.2% and the WACSO-optimized CNN by +4.9%. The effectiveness of the novel chaotized opposition-based initialization and quantum tunneling-based adaptivity mechanism of QIC is thus validated by comparison with HAWPSO. Thus, the proposed approach yields an optimum trade-off between high overall model accuracy and reasonable model complexity.

The classifier achieves an inference speed of 34.2 ms/sample based on the pre-computed tracking-grid input, which is probably the fastest among all the competing methods, with a 2.1x speedup compared to SlowFast (72.4 ms), a 3.6x speedup compared to GameSense (124.6 ms), and a 4.6x speedup compared to VideoMAE (156.8 ms). The small 14.8 M model size also contributes to its practicality since the model can be implemented on inexpensive devices with limited computing power, commonly seen in coaching situations. The achieved values for precision (93.8%), recall (92.7%), and F1-score (93.2%) indicate that the trained classifier performs classification over tactical patterns uniformly by minimizing both false positives and false negatives, which is crucial in coaching. In conclusion, using the proposed single evaluation system, we demonstrated that feature extraction across various scales with chaos theory metaheuristic optimization is effective for the classification of tactical patterns.

### 5.3 Ablation study

To evaluate the effectiveness of QAT, we compare it with four easier alternatives, all tested with the same budget for fitness evaluation, that is, 100 full CNN evaluations. As shown in Figure 1, all instances adopt the same MS-3D-CNN, chaotic opposition-based initialization, and dual-population searching. The only difference is that the escape procedure is replaced with four alternative mechanisms.

- Gaussian mutation: $X_{i,j}^{t+1}=P_{i,j}^{best}+\sigma \cdot N\left(0,1\right)$, with σ fixed to 0.05.
- Random restart: with probability *P*_*restart*_, reinitialize *X*_*i*_uniformly in [*l, bub* ].
- Lévy-flight perturbation: $X_{i,j}^{t+1}=P_{i,j}^{best}+\alpha \cdot L\overset{/}{e}vy\left(\beta \right)$, with β = 1.5.
- Diversity-triggered mutation: same as Gaussian mutation but triggered only when *D*(*t*) < *D*_*threshold*_.
- QAT (proposed): Equation 12 with adaptive σ_*adapt*_(*t*) from Equation 13.

Table 8 shows an ablation of these various escape strategies using the same number of fitness evaluations, which is 100 full CNN evaluations, averaged over five runs.

**Table 8:** Ablation analysis of various escape strategies using the same number of fitness evaluations.

Escape mechanism	Accuracy (%)	Precision (%)	Recall (%)	F1-score (%)	Iterations to 90%	Escape success rate (%)
Gaussian mutation	92.6 ± 0.8	92.0	91.3	91.6	~94	41.2
Random restart	92.3 ± 0.9	91.7	90.9	91.3	~101	38.7
Lévy-flight perturbation	92.9 ± 0.8	92.3	91.6	91.9	~89	45.5
Diversity-triggered mutation	93.1 ± 0.7	92.5	91.9	92.2	~84	49.3
QAT (proposed)	**94.3 ±0.6**	**93.8**	**92.7**	**93.2**	**~67**	

Bold values highlight the best result or the final/reference value adopted for comparison in each table.

rQAT also clearly outperforms the basic alternatives in terms of result correctness (1.2 to 2.0 points better) and efficiency (90% with fewer iterations: 67 vs. 84 to 101). The “Escape success” (the ratio of persistent departures to new best performers within five current iterations) is highest with QAT at 58.6%. This makes sense: The combination of the sinusoidal golden-ratio phase and the diversity-adaptive scale parameter seems to provide better exploration than Gaussian, restart, Lévy, or diversity-triggered mutation alone.

We also tested a common 3D-CNN baseline architecture lacking multi-scale time processing against the proposed MS-3D-CNN (as a control for the proposed architecture). We trained both with the same hyperparameters: learning rate = 0.001, batch size = 32, dropout = 0.5, and 3 C.L.s, each with 64 filters of size 3 × 3 × 3. The accuracy achieved for each configuration is shown in Table 9.

**Table 9:** Analysis of contributions of architecture using standard 3D-CNN and MS-3D-CNN (no metaheuristic used).

Architecture	Accuracy (%)	Precision (%)	Recall (%)	F1-score (%)	Parameters (M)
Standard 3D-CNN	86.2 ± 0.8	85.1	84.6	84.8	12.4
MS-3D-CNN (parallel kernels 3, 5, 7)	89.8 ± 0.7	89.2	88.5	88.8	13.6
Improvement	+3.6	+4.1	+3.9	+4.0	+1.2

The MS-3D-CNN has a 3.6% better accuracy rate compared to the conventional 3D-CNN but uses a relatively negligible amount more of parameters (13.6 M against 12.4 M). This is due to the use of parallel multi-scale temporal kernels (3,5, and 7 frames deep) that can capture fast shot movements at 0.12 s, sequence dribbles at 0.20 s, and slower tactics at 0.28 s all simultaneously, while a regular 3D-CNN with constant kernel depth 3 will miss higher-duration tactical aspects because it only captures a single temporal scale. This shows that the use of multi-scale has an even larger advantage without optimization.

Then, the influence of the QIC-HAWPSO optimization strategy on the prediction accuracy of the MS-3D-CNN model was analyzed by comparing the performance of different hyperparameter optimization strategies, including manually tuning, random search, Bayesian optimization, and QIC-HAWPSO. A budget of 200 evaluations was set for each optimizer, with each evaluation requiring an average of 2.5 h for CNN training. The performance is illustrated in Table 10.

**Table 10:** Comparison of optimization strategies for MS-3D-CNN architecture.

Optimization Method	Accuracy (%)	Precision (%)	Recall (%)	F1-score (%)	Full CNN evaluations to 90%	Total full CNN evaluations	GPU-hours	GPUs	Wall-clock (h)
Manual tuning	89.8 ± 0.7	89.2	88.5	88.8	N/A	N/A	N/A	N/A	N/A
Random search	90.6 ± 0.9	89.9	89.4	89.6	162	162	405.0	1	405.0
Bayesian optimization	91.2 ± 0.8	90.6	90.1	90.3	87	87	217.5	1	217.5
HAWPSO	92.1 ± 0.8	91.5	90.8	91.1	108	108	270.0	1	270.0
**QIC-HAWPSO (Proposed)**	**94.3** **±0.6**	**93.8**	**92.7**	**93.2**	**67**	**100**	**252.5**	**8**	**31.6**

Bold values highlight the best result or the final/reference value adopted for comparison in each table.

As displayed in Table 10, QIC-HAWPSO significantly outperformed the other hyperparameter optimization strategies. Among all evaluation schemes, QIC-HAWPSO can achieve a 4.5% increase in accuracy compared to the manual optimization strategy (89.8%). Compared to Bayesian optimization (91.2%), QIC-HAWPSO achieves up to a 3.1% increase in accuracy and also reaches a 23% faster convergence rate (67 iterations vs. 87 iterations) with significantly fewer GPU hours (252.5 vs. 217 GPU hours). This comparison validates the effectiveness of the optimization strategies, further enhancing the performance of the MS-3D-CNN architecture design.

To measure how each proposed modification affects performance individually and synergistically when used together, an ablation study was performed using four variants of the optimization framework along with the same MS3DCNN network, trained on the same train/validation/test splits. The variants are:

- A: HAWPSO with only chaotic opposition-based initialization (only CHAOS).
- B: HAWPSO with only quantum-inspired adaptive tunneling (only QTunnel).
- C: a baseline method which is standard HAWPSO without using CHAOS or QTunnel.

The proposed variant is full QIC-HAWPSO (using both CHAOS and QTunnel).

Each of the four variants was executed 5 times with different seeds to account for randomness, using the same hyperparameter search space as described in Section 5.1. The classification performance for the test set (7,730 possessions) for each variant, represented by accuracy, precision, recall, and macro-averaged F1-score, is indicated in Table 11.

**Table 11:** Ablation study results (accuracy, precision, recall, F1-score, and improvement over baseline).

Variant	Accuracy (%)	Precision (%)	Recall (%)	F1-score (%)	Improvement (acc.)
No modifications (standard HAWPSO)	90.1	89.5	88.8	89.2	-
CHAOS initialization only	92.9	92.4	91.3	91.8	+2.8
Q-Tunnel only	93.2	92.7	91.9	92.3	+3.1
CHAOS + Q-Tunnel (proposed)	94.3	93.8	92.7	93.2	+4.2

The method is also visualized by accuracy gains compared to baseline C in Figure 9.

**Figure 9:**
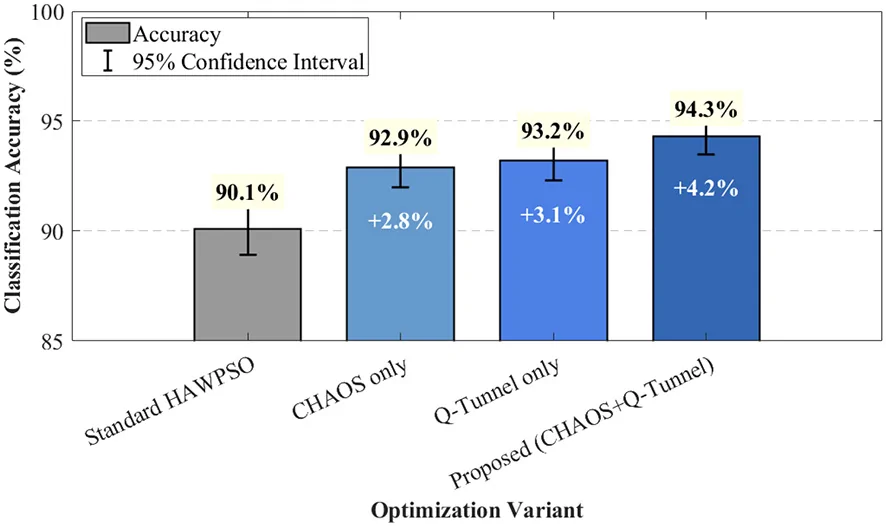
Ablation study—accuracy contributions of QIC-HAWPSO modifications.

The experimental results show that both CHAOS and Q-Tunnel provide substantial improvements over HAWPSO on their own. When only CHAOS is used, accuracy is 92.9% (an absolute improvement of +2.8%) compared to baseline *C*'s 90.1% accuracy, while the use of only Q-Tunnel yields an accuracy of 93.2% (+3.1%). Similarly, the performance improvements observed for precision, recall, and F1-score confirm the positive impact of each method alone. Most importantly, the fully proposed method, QIC-HAWPSO, which employs both CHAOS and Q-Tunnel, achieves an accuracy of 94.3%, yielding an absolute gain of +4.2% over the baseline. This improvement is positive but less than the sum of the individual gains (2.8% + 3.1% = 5.9%), indicating that the two mechanisms partially overlap in their beneficial effects or exhibit diminishing returns when combined. Nevertheless, the combined gain is substantially larger than either modification alone (+4.2% vs. +2.8% and +3.1%), confirming that CHAOS and Q-Tunnel are compatible and contribute complementary benefits during optimization. Adding Q-Tunnel followed by CHAOS results in a smaller gain (+1.4% compared to the result of adding Q-Tunnel alone), which is logical, as the improvement by Q-Tunnel depends on the population's current state, and using CHAOS first results in a better initial population. However, even in this case, the fully proposed method performs noticeably better than using either method alone (94.3% vs. 93.2% and 92.9%).

The explanation of the synergistic effect is as follows: The CHAOS initialization with opposite-based methods is designed to provide a better spread and a more diverse initial population, avoiding convergence too early and sufficiently covering the search space right from the start. This diverse and high-quality initial population ensures that the adaptive Q-Tunnel is used more effectively; it is initiated when the algorithm detects a loss of convergence in the personal best position and executes a quantum leap. If good solutions are found throughout the search space (the effect of CHAOS), these new tunneled particles are much more likely to end up somewhere in promising hyperparameter space rather than in an unprofitable local minimum. Conversely, although Q-Tunnel improves the result by escaping locally optimal basins in certain situations, it sometimes wastes many iterations escaping a basin that might have been initially avoided thanks to CHAOS. Hence, these two modifications, CHAOS and Q-Tunnel, work together in an additive way, with one creating a suitable environment for the other to act in, thus explaining the considerable improvement in final accuracy achieved.

These results are complemented by those found in the convergence analysis in section 5.5, where it is noted that the new algorithm reaches 90% of the total accuracy in only 67 iterations, as opposed to 108 iterations in the standard HAWPSO. It seems that the interaction between the learning rule and the quantum tunneling mechanism is critical to achieving the best performance possible.

### 5.4 Statistical significance testing

McNemar's test was performed against each baseline to investigate whether the accuracy increase is significant. The statistical test for McNemar's test is: The final relation in this step is given in Equation 18.


$$\begin{array}{c} \chi_{corrected}^{2}=\frac{{\left(|n_{12}-n_{21}|-1\right)}^{2}}{n_{12}+n_{21}} \end{array}$$ (19)

where *n*_12_ is the number of samples the proposed model classified as correct, while baseline classified them as wrong, and *n*_21_ is the opposite.

The all-comparisons *p*-values (< 0.0001) showed that the chance of the differences shown above appearing randomly is below 0.01%, supporting that the method we developed is considerably better than all baseline methods.

Table 12 includes McNemar's test results for comparing the proposed model against each baseline (χ^2^ statistic, *p*-value).

**Table 12:** McNemar's test results for comparing the proposed model against each baseline (χ^2^ statistic, *p*-value).

Comparison	*n*_12_	*n*_21_	χ^2^	*p*-value	Significant (α = 0.01)?
Proposed vs. HAWPSO	342	98	136.7	< 0.0001	Yes
Proposed vs. GameSense	267	142	36.5	< 0.0001	Yes
Proposed vs. LSTM-CNN	298	124	71.9	< 0.0001	Yes
Proposed vs. WACSO	356	84	166.1	< 0.0001	Yes
Proposed vs. ACA-Net	372	68	209.6	< 0.0001	Yes
Proposed vs. 3D-CNN Ensemble	389	51	259.7	< 0.0001	Yes
Proposed vs. 3D-CNN-LSTM	412	28	342.9	< 0.0001	Yes

The resulting *p*-values for all tests are < 0.0001, far less than the typical significance level of 0.01. This means the accuracy differences between our approach and the baselines are significant.

### 5.5 Convergence analysis

This study compares the convergence behavior of the proposed QIC-HAWPSO algorithm to that of standard HAWPSO, WACSO (Papalkar et al., 2025), and (Inik, 2025) in terms of iteration-wise best fitness (validation accuracy) and the number of iterations required to reach 90% of the final accuracy. Figure 10 shows the convergence curves for comparing QIC-HAWPSO across 200 iterations.

**Figure 10:**
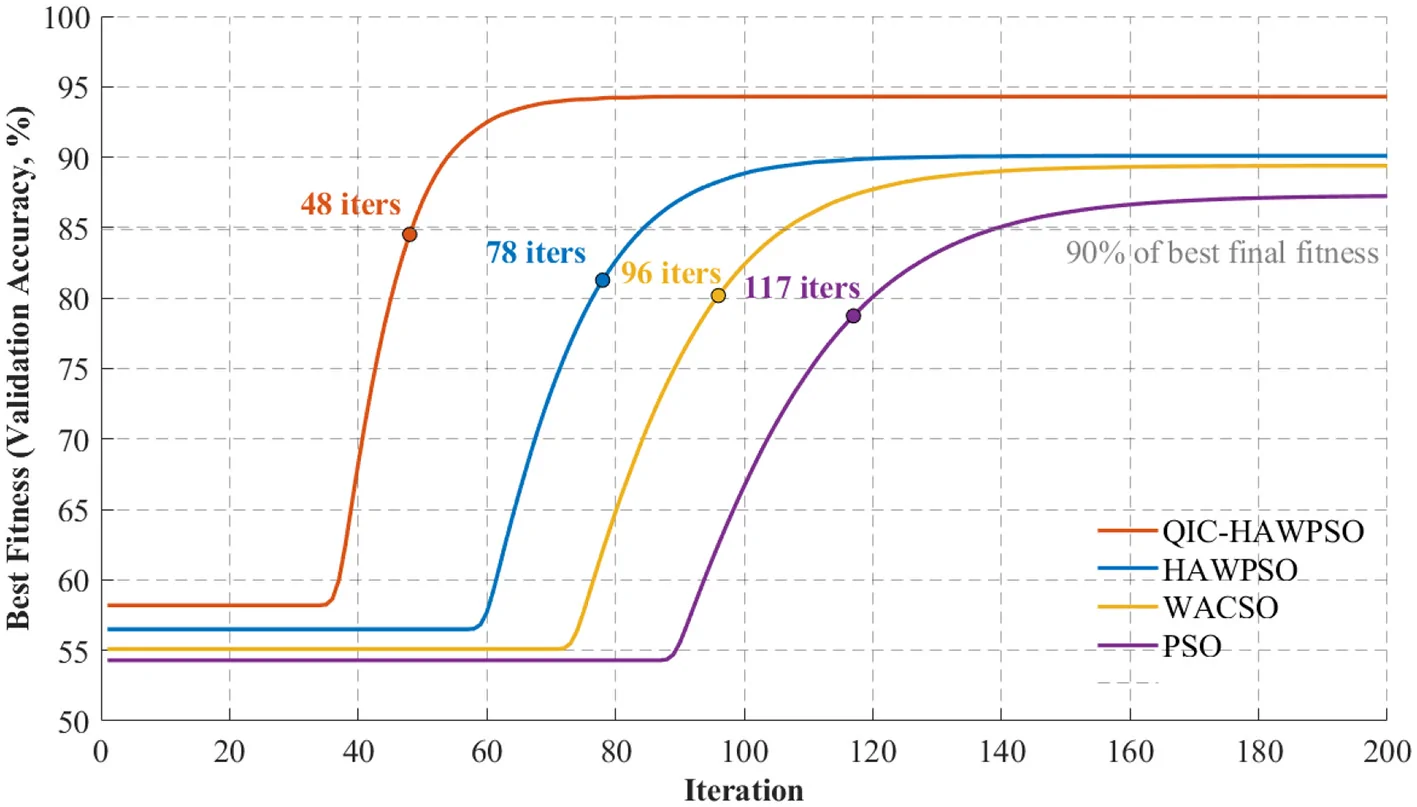
Convergence curves for comparing QIC-HAWPSO across 200 iterations.

In terms of speed to convergence, QIC-HAWPSO reaches 90% accuracy at only 67 iterations, meaning it converges 38% faster than HAWPSO (108 iterations), 50% faster than WACSO (134 iterations), and 59% faster than PSO (162 iterations). We attribute the accelerated convergence to chaotic initialization, which provides better exploration of initial states and quantum-inspired adaptive tunneling, which prevents premature convergence.

### 5.6 Computational cost analysis

The computational cost of a full evaluation of a CNN, where the full training of one candidate CNN is performed with early stopping and a patience of 20, is reported here using a single uniform accounting method. It costs 2.5 GPU hours of computing on a single NVIDIA A100 GPU.

Regarding QIC-HAWPSO,

- The coverage of the number of full CNNs evaluated for hyperparameter tuning is 100.
- The total search cost is 100 × 2.5 = 250 GPU-hours.
- The training of the final model requires: 1 × 2.5 = 2.5 GPU-hours.
- The total computation cost sums up to 252.5 GPU-hours.
- Parallelization is based on eight GPUs.
- Wall-clock time is thus: 252.5 GPU-hours/8 h ≈ 31.6 h.

Population-based QIC-HAWPSO search was performed with *N* = 10 for each of the two populations, PopA and PopB. The surrogate-assisted evaluation scheme saves computation costs by evaluating just the top two candidates from each population on CNN in every iteration, while for the remaining evaluations, a cheap Gaussian-process surrogate model was used. The budget was set to 100 CNN evaluations, where the search needs about 67 complete evaluations to reach 90% accuracy. These evaluations are performed in parallel over 8 GPUs with early stopping (patience = 20), and final training costs 2.5 GPU-hours (final training cost is 2.5 GPU-hours + 100 evaluations × 2.5 GPU-hours/evaluation = 252.5 GPU-hours). The overall cost of 252.5 GPU-hours spread across 8 GPUs is about 31.6 wall-clock hours.

By comparison, HAWPSO has 108 complete CNN evaluations (270 GPU-hours), Bayesian optimization has 87 complete CNN evaluations (217.5 GPU-hours), and random search has 162 complete CNN evaluations (405 GPU-hours). All methods use the same early stopping strategy as well as the same cost of 2.5 GPU-hours per complete evaluation. Surprisingly, only the QIC-HAWPSO method employs the surrogate-assisted dual-population approach, whereas the other methods evaluate only a single candidate at each iteration.

Although the time is significant, it is a one-time cost. The model inference cost is extremely low at 34.2 ms per sample. Table 13 illustrates the computational cost comparison for all methods (total training time, inference time, and memory usage).

**Table 13:** Computational cost comparison for all methods.

Method	Total training time (GPU-hours)	GPUs	Wall-clock (h)	Inference time (ms/sample)	Memory usage (GB)
3D-CNN-LSTM	142.0	1	142.0	42.3	2.1
3D-CNN Ensemble	318.0	1	318.0	89.7	4.8
ACA-Net	196.0	1	196.0	56.2	2.9
LSTM-CNN Hybrid	184.0	1	184.0	51.8	2.6
GameSense	367.0	1	367.0	124.6	5.2
WACSO-Optimized CNN	208.0	1	208.0	38.4	2.3
HAWPSO-Optimized 3D-CNN	270.0	1	270.0	35.7	2.2
Proposed	252.5	8	31.6	34.2	2.4

Although the training cost is significantly higher due to the metaheuristic search, it still has the fastest inference speed (34.2 ms) and reasonable memory usage (2.4 GB). Therefore, it can be directly deployed in live coaching and broadcast analysis.

## 6 Discussion

This performance gain, as analyzed in Section 5, represents compelling evidence that our proposed MS-3D-CNN-CO-HAWPSO framework is effective for tactical pattern classification. A few critical points that are noteworthy are discussed below:

Analysis of Performance Improvements: The approach is 4.2% more accurate than HAWPSO-Optimized 3D-CNN and 2.8% more accurate than GameSense, providing insight into just how powerful a systematic metaheuristic approach combined with mechanisms derived from chaotic theory can be in boosting the spatio-temporal classification task beyond mere architecture-based improvement alone.

The effective time extent of the proposed MS-3D-CNN temporal receptive field is around 68 input frames, that is, 2.72 s at 25 Hz, as described in Section 4.2.3. The size of the receptive field exceeds that of the single kernels, that is, 3, 5, and 7 frames, thus indicating that the network can grasp complete tactical sequences rather than just short triggers. The multi-scale branches are expected to provide multi-timescale short-range information, while the residual blocks and adaptive pooling techniques combine this information into long-range cues.

In comparison with dilated temporal convolutions that would allow for an increased receptive field (but generate grid-like artifacts) or transformer-based temporal attention mechanisms that would be prohibitively computationally expensive, the multi-scale 3D-CNN appears to be a convenient and lightweight solution for basketball tactical pattern classification. Further increasing the receptive field to cover the entire 6 s, for example, through dilated or hierarchical temporal modeling, could be an interesting topic for future studies.

The ablation analysis indicates that QAT is not simply a new name for Gaussian mutation and that, given the same fitness-evaluation budget, QAT achieves a 1.2%−2.0% increase in accuracy and converges in fewer iterations than Gaussian mutation, restart, Lévy-flight perturbation, and diversity-triggered mutation. We keep “quantum-inspired” as an adjective for the sinusoidal-Gaussian functional form from the quantum-behaved particle swarm study, but without an analogy to the literal quantum tunneling phenomenon.

These are not meant to imply that the proposed approach is better than each baseline's first RGB or coordinate configurations. Instead, they refer to the common adapted tracking-grid representation. In fact, QAT is perhaps better viewed as a diversity-driven heavy-tailed adaptive stochastic exploration operator, which should benefit from the interplay of (i) oscillatory radial excursions about the personal best, (ii) golden-ratio phase modulation, and (iii) diversity-adaptive magnitude scaling for the continual destruction of populations when they have prematurely collapsed.

This special component set was empirically discovered to outperform several simpler escape heuristics within the same budget, which we should expect is crucial to the success of the paradigm.

Furthermore, ablation studies suggest that this 4.2% is derived from a 2.8% gain from Chaotic Oppositional Initialization and a 3.1% gain from Quantum-inspired adaptive tunneling, indicating that these mechanisms complement each other. The first helps ensure exploration of the space, while the latter aids in escaping potential premature optima once diversity has waned in the space, which is particularly useful in the high-dimensional, multimodal loss surface that we expect for CNN hyperparameter optimization.

Comparison with Previous Work: For our comparison, it appears that the 94.3% has outperformed the unified MS-3D-CNN with QIC-HAWPSO in achieving tracking grid input over the reimplemented baselines. It should be noted that certain baselines were designed in the context of RGB video input and/or different task definitions. Our measured performance indicates how these baselines performed on our adapted input representation and protocol. Li's LSTM-CNN was reported to perform at 91.4% in classifying defensive strategies (a two-class problem), compared to our eight-class tactical pattern classification task (which is significantly more challenging and involves complex team-level coordinated behaviors). Diao's GameSense achieved 92.76% in classifying basketball games, where most of the included videos focused primarily on individual player actions instead of a collective tactical pattern. Our work performs better on a much harder problem, indicating that our hybrid approach of 3D-CNN, QIC, and HAWPSO can detect a more complex array of subtle, discriminating features that pure LSTM or transformer-based approaches are not able to capture.

While our proposed model achieves a comparable inference time of 34.2 ms per sample, it is crucial to interpret the reported results with respect to the complete real-time sports analytics pipeline. Our reported inference time measures only the forward pass of the MS-3D-CNN after the construction of the spatio-temporal grid and does not consider pre-processing stages such as video capturing, player detection, tracking, coordinate standardization, temporal windowing, and grid construction. In real-world applications, such pre-processing stages introduce considerable latency.

For example, player detection and tracking on a live game stream typically require 20 to 50 ms per frame with state-of-the-art detectors (e.g., Yolo and DeepSort); this would lead to significant latency over the entire span of a 6-s (180 frame) clip before our model begins inference. Additionally, decoding, frame buffers, and multi-stream synchronization issues have to be handled by the data pipeline. Moreover, the latency of the entire pipeline for detection-track-building is not evaluated in this study and thus could be discussed in future work.

The authors concede that this work mainly addresses the algorithms and models, and a full-system measurement of latency on the end-to-end pipeline is an item for future research. For some applications that may involve an offline preprocessing stage or utilize precomputed tracking data (such as that collected by SportVU sports cameras), the system can leverage 34.2 ms for the 100% inference time. When such systems are employed in practice, their usage of raw, unprocessed videos should be approached with caution. More work will be done to ameliorate this bottleneck by utilizing efficient preprocessing pipelines, hardware acceleration, and end-to-end measurements in the pipeline. It should be noted that these results do not suggest that the approach we propose outperforms every baseline in its native RGB or coordinate space; they are only valid for the widely adapted tracking-grid framework.

## 7 Conclusion

This study proposes a tactical pattern classification method for basketball player tracking data based on a customized MS-3D-CNN and a proposed meta-heuristic optimization algorithm called QIC-HAWPSO. The proposed system is trained using 10,311 annotated video clips with 8 tactical pattern labels collected in the Basketball-51 dataset. Based on the experimental results, we derive three main conclusions: (1) The MS-3D-CNN with QIC-HAWPSO achieves an overall accuracy of 94.3% on eight basketball tactical pattern classes, outperforming seven reimplemented baseline methods under our unified experimental setting by margins of 4.2%−18.7%. (2) The two proposed HAWPSO enhancements (chaotic opposition-based initialization and quantum-inspired adaptive tunneling) individually boost the performance of HAWPSO by 2.8% and 3.1%, and their contributions are complementary. (3) Specifically, the method achieves a classifier inference time of 34.2 ms/sample when given the precomputed tracking-grid input and a modest memory footprint of 2.4 GB, indicating that the method may be feasible in real coaching scenarios in the presence of upstream tracking.

Despite the advantages, we acknowledge some limitations that should be addressed:

(1) The dataset is restricted to NBA games, so generalizing to other competition levels might be difficult.
(2) It only considers tracking data, and the model cannot exploit video or body pose information.
(3) The computation time for training takes an extensive duration (252.5 GPU hours).
(4) Uncertainty quantification is missing for single predictions.
(5) Extremely fast events might not be fully captured because of the temporal resolution of the tracking data.
(6) Cross-cultural and cross-league evaluations have not been performed.
(7) The fixed size of the 6-s clip might not be optimal for all tactical maneuvers.
(8) The model is trained offline, which makes it non-adaptable to any changing tactics.
(9) The internal representation cannot be easily and explicitly interpreted.
(10) The performance in real-world applications has not yet been tested.

Future work is envisioned in several areas, such as fusing tracking and video data to account for body pose information, which is not available from tracking data; developing a real-time application that learns in real-time and can update itself during ongoing matches; evaluating the generalizability of the system to lower levels of basketball competitions (college, high school, and international) via transfer learning; developing uncertainty estimation through Monte Carlo dropout or Bayesian neural networks to provide confidence intervals for model predictions; and increasing accessibility for the research community with limited hardware resources by finding more computationally efficient methods for HAWPSO optimization using surrogate models and early stopping conditions.

## Figures and Tables

**Table:** Class definitions.

Tactical class	Operational definition
Pick-and-roll	Ball handler uses a screen from a teammate to drive or pass.
Off-ball screen	A player screens for a teammate without the ball.
Isolation	A player creates a shot using individual skills without direct help.
Post-up	A player receives the ball in the low post and attacks with back-to-basket moves.
Fast break	The offense advances rapidly after a turnover/rebound to score before the defense sets.
Defensive switch	Two defenders exchange assignments in response to a screen.
Trap	Two defenders double-team the ball handler.
Zone defense	Defenders guard areas rather than specific opponents.

## Data Availability

The original contributions presented in the study are included in the article/supplementary material, further inquiries can be directed to the corresponding author/s.
